# Association of Use of Electronic Appointment Reminders With Waiting Times in the Veterans Affairs Health System

**DOI:** 10.1001/jamanetworkopen.2021.48593

**Published:** 2022-02-15

**Authors:** Lianjun Li, Haiqing Zhao, Noah Lim, Joel Goh, Bernard Ng

**Affiliations:** 1 Global Asia Institute, National University of Singapore, Singapore; 2 Department of Analytics and Operations, NUS Business School, National University of Singapore, Global Asia Institute, Singapore; 3 Technology and Operations Management Unit, Harvard Business School, Boston, Massachusetts; 4 Department of Medicine, VA Puget Sound Health Care System, Seattle, Washington; 5 Rheumatology Section, Department of Medicine, University of Washington, Seattle

## Abstract

**Importance:**

Electronic appointment reminder systems are increasingly used across health systems. However, their association with patients’ waiting times for their appointments, a measure of timely access to care, has yet to be assessed.

**Objective:**

To assess the associations between the introduction of an electronic appointment reminder system and the number of days patients had to wait from appointment booking to appointment completion in patients in the Veterans Affairs Health System.

**Design, Setting, and Participants:**

Cohort study of patients who completed appointments from January 1, 2018, to October 13, 2018, inclusive in all 130 Veterans Affairs (VA) health centers in the US. The study population comprised a census of all patients who received care at any VA health center during the period of the study for outpatient, procedural, rehabilitation, or radiology services. Data were analyzed from May 15, 2021, to December 15, 2021.

**Exposures:**

Phased introduction of an electronic appointment reminder system (VEText) in 6 waves spread across the study period.

**Main Outcomes and Measures:**

The unit of observation in this study was a completed appointment made by any such patients. Observations were excluded if the appointment was booked before but completed after the exposure, or if data were duplicated, missing, or incomplete. For each completed appointment, the number of days between which the appointment was booked and when it was completed.

**Results:**

The number of observations after exclusion comprised 39.5 million completed appointments from 5.1 million patients (91.1% male) with a mean (SD) age of 62.57 (16.24) years. The adoption of VEText was associated with an estimated reduction in patient waiting time by a mean of 6.51 days (95% CI, 5.51-7.52 days). Adoption of VEText was also associated with an increase of 8.54 (95% CI, 7.65-9.44) days of additional waiting per incomplete booking.

**Conclusions and Relevance:**

Results of this study suggest that appointment reminder systems may be associated with decreases in the mean number of days patients in the VA system have to wait for their appointments but can potentially lengthen waiting times for patients who miss their bookings. Further study is warranted to assess whether these findings may be generalizable to other populations.

## Key Points

**Question:**

Is use of an electronic appointment reminder system associated with the number of days patients have to wait to complete their appointments?

**Findings:**

In this cohort study of 5 116 085 patients and 102 249 484 bookings in the Veterans Affairs Health System, the introduction of an electronic appointment reminder system was associated with a mean reduction in waiting time of 6.51 days compared with a baseline wait of approximately 60 days.

**Meaning:**

Results of this study suggest that use of an electronic appointment reminder system may be associated with improved patient access to health services.

## Teaser

This cohort study assesses the association between the introduction of an electronic appointment reminder system and the number of days from appointment booking to appointment completion among patients who received care in the Veterans Affairs Health System.

## Introduction

Electronic appointment reminder systems are increasingly being used across health systems in the US and around the world. Their prominence may be associated with their modest (and decreasing) costs of implementation, as well as their association with improvement in patient no-show rates.^1,2,3,4,5,6^

Although there has been much investigation into associations between such reminder systems and patients’ appointment-keeping behavior, research into use of these systems and patient waiting times (the number of days from when an appointment is booked to when the patient completes the appointment) is limited. Waiting times capture patients’ delay in receiving care and are an important measure of a health care delivery system’s responsiveness. They also indirectly measure patient access, an indicator that is known to be associated with clinical outcomes.^7,8,9^

It is worth noting that the positive findings of appointment reminder systems regarding no-show rates do not immediately translate into improved waiting times. On the one hand, a lower no-show rate means that fewer patients miss their appointments and more patients receive care expediently. On the other hand, a higher influx of patients stemming from lower no-show rates could be a factor in more congested appointment schedules, which could inadvertently increase the length of time between when patients book their appointments and when they eventually receive care. In addition, some studies have found that reminder systems, implemented either through telephone calls or cellphone text messages, may be associated with higher rates of appointment cancellation.^10,11,12,13^ Increased rates of cancellation mean that patients (intentionally or otherwise) may experience delay in receipt of their care, and that congestion of clinic waiting lists and patient waiting times may increase.

A systematic investigation into the association between appointment reminder systems and patient waiting times has, to our knowledge, not been undertaken. The present study aims to take a first step toward bridging this gap in the context of the VA health care system, which is the largest integrated health system in the US. In 2018, the VA health system rolled out an electronic appointment reminder system (VEText) across its medical centers and outpatient facilities in the US. Clinics adopting the VEText system would send out 2 text message reminders to patients’ mobile phones before their appointment. The modal number of days for the first reminder was 7 days (87.87% of clinics) prior to patients’ appointments. For the second reminder, 80.02% of clinics sent this reminder out between 1 and 3 days prior to patients’ appointments. Before VEText, the VA health system did not have a central system for appointment reminders; some clinics used telephone reminders, some used reminder letters, and others did not use reminders. The rollout of VEText was staggered across 6 separate waves (initiation dates of these waves were March 7, March 21, April 4, April 18, May 2, and May 16 in 2018). This staggered rollout forms the basis for the cohort research design of this study, allowing us to estimate the outcomes associated with the VEText introduction and each patient’s waiting time.

## Methods

This study was approved by the Office of Research and Development, Department of Veterans Affairs with a waiver of informed consent because the study posed negligible risk to patients, would not adversely affect their welfare, and the large scale of this study made it infeasible to conduct without this waiver. This study followed the Strengthening the Reporting of Observational Studies in Epidemiology (STROBE) reporting guideline for observational studies.

Our data set comprised all appointments booked by patients at any 1 of 130 VA health care facilities across the US for an appointment date that occurred between January 1, 2018, and October 13, 2018. This range comprised 102 249 484 bookings. Data were analyzed from May 15, 2021, to December 15, 2021.

This study focuses on the subset of bookings that resulted in completed appointments. The primary outcome metric was the patient’s waiting time defined as the number of days from the date that a patient first books their appointment (booking date) to the date that the patient eventually sees their clinician and completes the appointment (appointment date). This metric was chosen as a representation of how quickly patients actually receive care. A longer waiting time entails a greater delay in a patient’s receipt of care and may be a sign of reduced patient access. Between patients’ booking and appointment dates, they may have 1 or more incomplete bookings (cancellations or no-shows) for appointments. Incomplete bookings potentially lengthen patients’ waiting times; hence, we recorded and controlled for the number of such incomplete bookings prior to each completed appointment.

We restricted the data to completed appointments from 15 classes, which represented different medical specialties or types of care that were further categorized into 4 distinct clinical groups: regular outpatient (medical clinics, surgical clinics, mental health clinics, pharmacy, nutrition, dental, rehabilitation clinic, spinal cord, and substance use), procedural (medical procedure, laboratory procedure, surgical procedure, and mental health procedure), rehabilitation therapy, and radiology. We also excluded any appointments that were duplicated, had missing or erroneous data (eg, booking dates after appointment dates). In addition, we excluded all appointments for patient-class combinations with 19 or more completed appointments in the data set (top 1%) because we expected that patients in the top 1% of visit rates for a given medical class could have health conditions and care needs that were outliers and not representative of the broader VA patient population.

The intervention indicator, the key independent variable, was whether a given completed appointment at a given clinic was made before or after the clinic adoption of the VEText reminder system. Adoption of VEText at a clinic is a separate notion from the 6 waves of VEText rollout(an instrumental variable) The control group comprised all completed appointments whose appointment dates preceded the adoption of VEText at their respective clinics. For these observations, the intervention indicator was set to 0. Conversely, the intervention group comprised all completed appointments whose appointment dates were after VEText was adopted and for which every patient had received at least 1 VEText reminder. For these observations, the intervention indicator was set to 1. Appointments for which VEText was adopted between booking and completion dates were considered indeterminate and were excluded.

Because each clinic could choose its own VEText adoption times, this self-selecting behavior may bias results from ordinary regression analyses that simply include the intervention indicator as an independent variable. Conceptually, this situation is often referred to as the problem of selection on unobservables, which in the specific context of this study, may occur because clinics could base their adoption decisions on information that is not captured in the data. One way to correct this bias is through instrumental variable regression analysis, a statistical approach^14,15^ that has been used in other studies with observational data.^16,17,18^ To conduct an instrumental variable analysis, an eponymous instrument, is required which is a new variable that has 2 defining features. First, the instrument must be correlated with the original intervention variable. Second, the instrument should not be affected by the same selection on unobservable problem that affect the original intervention variable.

For this study, we used the fact that the VEText rollout over 6 separate waves was coordinated by the VA Central Office. For each clinic, the VEText rollout represented a nonbinding guidance for clinics to adopt VEText. As could be expected, this introduction would be aligned with each clinic’s adoption decision but would not otherwise be a factor in their scheduling operations. Moreover, because the VEText rollout was centrally coordinated, it was not subject to the same self-selection issues as individual clinics’ decisions of VEText adoption. Therefore, it is unlikely that there were other unobserved variables through which the VEText rollout indicator was associated with waiting times. This rollout indicator therefore fulfills the 2 defining criteria of an instrumental variable.

### Statistical Analysis

We used regression models to assess the association between the introduction of VEText and patients’ waiting times and considered different model specifications. The simplest model (model 1) was a multivariable linear regression, (ie, multivariable ordinary least squares regression) which controlled for patient-level demographic confounders (eg, age, sex, self-reported race), appointment-level confounders (eg, day-of-week and month of the appointment date), as well as the facility and class of the appointment. Calendar month was modeled as a categorical variable to capture its potentially nonlinear association with waiting time. Complete descriptions of these variables are provided in eTable 1 in the Supplement. A second model (model 2) added 2 controls to model 1: the number of incomplete bookings prior to that appointment, and an interaction term that multiplied this variable with the intervention indicator. Models 3 and 4 were analogous to models 1 and 2 respectively, with the key exception being that they were instrumental variable regressions that used the rollout indicator as an instrument to correct for self-selection bias.

Post hoc analyses were conducted to assess associations between VEText and the number of patient cancelled bookings and the number of such bookings cancelled by patients at short notice (defined as cancellations within *t* days prior to the appointment date; we separately investigated the cases of *t = *7, *t = *14, and *t = *21). These analyses were instrumental variable regression models with the same structure of model 3 and appropriately modified dependent variables.

We performed subgroup analysis to investigate the differences in VEText and patient waiting times across the 4 clinical groups of regular outpatient, procedural, rehabilitation therapy, and radiology. We stratified the data set by clinical group and applied the regression analysis of model 4 within each stratum. We also conducted analyses to investigate the sensitivity of our results to alternative modeling assumptions and across further substrata of the data (eMethods in the Supplement).

We report point estimates of the regression coefficients along with robust SEs clustered at the clinic level.^14^ All statistical analyses were conducted using Stata/MP statistical software version 17.0 (StataCorp). Regression coefficients were labeled as statistically significant if their 95% CIs did not include 0. Full mathematical details of all regressions are reported in eMethods in the Supplement.

## Results

The final study sample comprised 39 488 685 observations (completed appointments) made by 5 116 085 individuals. A flowchart of the data construction process is depicted in eMethods in the Supplement. Summary statistics of the cohort are reported in Table 1. Patients had a mean (SD) age of 62.57 (16.24) years. Most patients were male (91.13%) and self-reported their race and ethnicity as White (78.11%). More than half (56.54%) of the patients were married. Before VEText, the mean (SD) waiting time was 53.79 (75.36) days compared with a mean (SD) of 60.69 (83.65) days after VEText.

**Table 1.** Description of Data Sample

Variable	Clinical group, No. (%)				
	Regular outpatient^a^	Procedural^b^	Rehabilitation therapy^c^	Radiology^d^	Overall
No. of unique patients	5 116 085	2 760 769	582 971	1 367 173	5 259 832
Age, mean (SD), y	62.67 (16.19)	63.15 (15.28)	62.34 (15.62)	62.39 (14.09)	62.57 (16.24)
Sex					
Male	91.13	91.54	88.56	87.73	91.10
Female	8.87	8.46	11.44	12.27	8.90
Race and ethnicity					
African American/Black	18.63	17.28	24.21	21.23	18.63
American Indian	1.02	1.01	1.06	0.99	1.02
Asian	1.22	0.85	1.30	0.91	1.21
Pacific Islander	1.02	0.98	1.11	0.97	1.02
White	78.11	79.87	72.33	75.90	78.12
Marital status					
Married	56.54	56.61	54.49	52.67	56.40
Divorced	22.08	23.16	23.82	26.20	22.13
Single	13.09	11.97	12.75	12.68	13.17
Waiting time, mean (SD), days					
Control group	58.68 (75.36)	53.36 (80.28)	20.06 (22.15)	28.74 (50.29)	53.79 (74.06)
Intervention group	64.64 (83.65)	63.09 (89.67)	19.49 (21.34)	28.66 (51.18)	60.69 (82.46)
No. of incomplete bookings, mean (SD)					
Control group	0.31 (0.72)	0.18 (0.53)	0.28 (0.73)	0.18 (0.53)	0.27 (0.68)
Intervention group	0.30 (0.72)	0.18 (0.51)	0.25 (0.71)	0.15 (0.49)	0.27 (0.68)
No. of observations					
Control group	12 566 377	3 520 376	763 353	1 363 313	18 213 419
Intervention group	15 984 362	3 337 951	942 034	1 010 919	21 275 266

^a^ Regular outpatient classes include medical clinics, surgical clinics, mental health clinics, pharmacy, nutrition, dental, rehabilitation clinic, spinal cord, and substance abuse.

^b^ Procedural classes include medical procedure, laboratory procedure, surgical procedure, and mental health procedure.

^c^ Rehabilitation therapy classes include rehabilitation therapy.

^d^ Radiology classes include radiology.

Table 2 reports the main results of our aggregate analyses across the 4 clinical groups. eTables 2 and 3 in the Supplement present full results. The coefficient of the intervention variable represents the number of days of additional waiting time associated with the introduction of VEText. From model 1, we observe that the introduction of VEText was associated with a mild but statistically significant reduction of mean patient waiting times by 3.23 (95% CI, 1.97-4.48) days. After controlling for self-selection (model 3), VEText was estimated to be associated with a substantial reduction in mean waiting time by 10.20 (95% CI, 9.20-11.20) days, which is a 19% reduction compared with the mean waiting time in the control group.

**Table 2.** Association Between Waiting Times and VEText Intervention With Different Regression Models^a^

Explanatory variable	Estimate (95% CI)			
	Linear regression		Instrumental variable regression	
	Model 1	Model 2	Model 3	Model 4
VEText intervention	−3.23 (−4.48 to −1.97)	−1.36 (−2.60 to −0.11)	−10.20 (−11.20 to −9.20)	−6.51 (−7.52 to −5.51)
No. of incomplete bookings	NA	26.25 (25.40 to 27.10)	NA	23.88 (23.11 to 24.66)
Intervention × (No. of incomplete bookings)	NA	4.33 (3.25 to 5.42)	NA	8.54 (7.65 to 9.44)
No. of observations	39 488 685	39 488 685	39 488 685	39 488 685

Abbreviations: NA, not applicable; VEText, electronic appointment reminder system via text messaging.

^a^ All results have been adjusted for the following patient-level confounders: age, age squared, sex, race, marital status, insurance status, calendar month of appointment date, day-of-week of appointment date, number of past appointments from January 1, 2018, number of distinct and appointment classes from January 1, 2018. The results also include adjustments for the following clinic-level confounders: number of completed appointments in the past week, total number of appointments in the past week, and total number of patients seen from January 1, 2018.

From the results of model 4, the association of VEText on waiting times can be decomposed into 2 components. First, VEText was associated with a reduction of 6.51 (95% CI, 5.51-7.52) days of waiting. Second, VEText modified the number of days of delay between incomplete bookings and waiting times. Before VEText, each incomplete booking was associated with an increase of 23.88 (95% CI, 23.11-24.66) days in waiting time. After VEText, each incomplete booking was associated with an additional 8.54 (95% CI, 7.65-9.44) days of waiting; after VEText, each incomplete booking was associated with a mean of 32.42 (95% CI, 31.28-33.58) days of delay per incomplete booking

The results of subgroup analysis for model 4 are reported in Table 3. We found that the association of VEText with waiting time varied between groups. Across clinical groups, the procedural group saw the largest reduction in waiting time associated with VEText, estimated at 10.24 (95% CI, 6.84-13.64) mean days. Moderate decreases were observed in the regular outpatient groups and radiology groups, which saw approximately a mean of 3 to 6 days of reduced waiting associated with VEText. Full results of these regressions are reported in eTable 4 in the Supplement.

**Table 3.** Association Between Waiting Times and VEText Intervention Using Model 4, Stratified by Clinical Group^a^

Explanatory variable	Clinical group, estimate (95% CI)			
	Regular outpatient	Procedural	Rehabilitation therapy	Radiology
VEText intervention	−5.73 (−7.04 to −4.41)	−10.24 (−13.64 to −6.84)	−1.52 (−2.17 to −0.86)	−3.31 (−4.98 to −1.64)
No. of incomplete bookings	25.21 (24.33 to 26.10)	22.73 (20.93 to 24.53)	9.34 (8.95 to 9.74)	15.71 (14.07 to 17.34)
Intervention × (No. of incomplete bookings)	7.80 (6.86 to 8.75)	15.15 (12.15 to 18.14)	2.32 (1.26 to 3.38)	16.62 (13.50 to 19.74)
No. of observations	28 550 739	6 858 327	1 705 387	2 374 232

Abbreviation: VEText, electronic appointment reminder system via text messaging.

^a^ All results have been adjusted for the following patient-level confounders: age, age squared, sex, race, marital status, insurance status, calendar month of appointment date, day-of-week of appointment date, number of past appointments from January 1, 2018, number of distinct and appointment classes from January 1, 2018. The results also include adjustments for the following clinic-level confounders: number of completed appointments in the past week, total number of appointments in the past week, and total number of patients seen from January 1, 2018.

Across all clinical groups, each incomplete booking was associated with an additional delay in receiving care, and VEText was associated with an exacerbation of this delay. However, the estimates varied across the clinical groups. For appointments in the procedural and radiology groups, VEText was associated with an additional delay of 15.15 (95% CI, 12.15-18.14) in the procedural group and 16.62 (95% CI, 13.50-19.74) in the radiology group per incomplete booking, approximately doubling the baseline delay associated with incomplete bookings. The coefficients of the interaction terms between incomplete bookings and waiting times were estimated as 7.80 (95% CI, 6.86-8.75) days per incomplete booking in the regular outpatient group and 2.32 (95% CI, 1.26-3.38) days per incomplete booking in the rehabilitation therapy group. Additional sensitivity analyses using alternative modeling assumptions and further subgroup analyses were consistent with these results (eTables 5, 6 in the Supplement).

Post hoc analysis results revealed that the introduction of VEText was associated with a reduction of 0.10 (95% CI, 0.10-0.10) cancelled bookings per completed appointment. Significant negative associations were also found within each of the 4 clinical groups, and for cancelled bookings at various definitions of short notice. For example, in the regular outpatient and procedural groups, the introduction of VEText was associated with a reduction of 0.11 (95% CI, 0.11 - 0.12) and 0.06 (95% CI, 0.05 - 0.07) cancelled bookings per completed appointment respectively (Table 4; eTables 7, 8, 9, 10 in the Supplement).

**Table 4.** Association Between Number of Cancellations and VEText Intervention, in Aggregate and Stratified by Clinical Group, for Different Dependent Variables^a^

Dependent variable	Clinical group, estimate (95% CI)				
	All clinical groups	Regular outpatient	Procedural	Rehabilitation therapy	Radiology
All cancellations	−0.10 (−0.10 to −0.10)	−0.11 (−0.12 to −0.11)	−0.06 (−0.07 to −0.05)	−0.06 (−0.07 to −0.05)	−0.06 (−0.07 to −0.05)
Cancellations within 21 d of appointment	−0.09 (−0.09 to −0.08)	−0.10 (−0.10 to −0.09)	−0.05 (−0.06 to −0.05)	−0.06 (−0.07 to −0.05)	−0.06 (−0.07 to −0.05)
Cancellations within 14 d of appointment	−0.08 (−0.09 to −0.08)	−0.09 (−0.10 to −0.09)	−0.05 (−0.06 to −0.04)	−0.06 (−0.07 to −0.05)	−0.06 (−0.07 to −0.05)
Cancellations within 7 d of appointment	−0.08 (−0.08 to −0.07)	−0.09 (−0.09 to −0.08)	−0.05 (−0.05 to −0.04)	−0.05 (−0.06 to −0.04)	−0.05 (−0.06 to −0.04)
No. of observations	39 488 685	28 550 739	6 858 327	1 705 387	2 374 232

Abbreviation: VEText, electronic appointment reminder system via text messaging.

^a^ All results have been adjusted for the following patient-level confounders: age, age squared, sex, race, marital status, insurance status, calendar month of appointment date, day-of-week of appointment date, number of past appointments from January 1, 2018, number of distinct and appointment classes from January 1, 2018. The results above also include adjustments for the following clinic-level confounders: number of completed appointments in the past week, total number of appointments in the past week, total number of patients seen from January 1, 2018.

To better understand the possible mechanisms of these results, we conducted further analyses that compared the mean daily workload before and after VEText, measured by the mean number of completed appointments at each clinic per day. Before VEText, each clinic had a mean of 22.2 (95% CI, 22.1-22.3) completed appointments per day; after VEText, mean workload at each clinic was significantly higher at 30.5 (95% CI, 30.3-30.6) completed appointments per day. Although not fully conclusive, this is nonetheless consistent with the notion that appointment schedules after VEText tended to be more tightly packed than before.

## Discussion

Previous studies have demonstrated beneficial outcomes associated with electronic appointment reminder systems and individual patient appointment-keeping behavior.^1,2,3,4,5,6^ The present study investigates whether such reminder systems are associated with improved measures of health care delivery, focusing specifically on reductions in patient waiting times.

Using data from the VEText reminder system, we found an association between reminder systems and measures of health care delivery, both in aggregate and separately for each of the 4 clinical groups. We also found that each incomplete booking prior to the completed appointment was associated with a concomitant and substantial increase in mean waiting times. This result may be expected; a patient who does not complete their appointment booking either through a cancellation or no-show would have to make a booking for a new appointment, which should entail additional delay.

The results also revealed that the introduction of VEText was associated was an exacerbation of the delay from incomplete bookings. We found evidence of some degree of exacerbation in each of the clinical groups, although this exacerbation was more pronounced in the procedural and radiology groups compared with others.

The results of our post-hoc analyses present a possible explanation for this apparent exacerbation. VEText was associated with a reduction in the number of patient cancellations and short-notice cancellations. When clinics receive such cancellations, they may not be able to find other patients to fill these newly open slots. Therefore, without VEText, higher levels of cancellations could mean that clinic appointment schedules may be more loosely filled. Conversely, because VEText was associated with fewer cancellations, under VEText, clinics may have appointment schedules with fewer gaps. In this case, a patient who had just missed their booking and was attempting to rebook an appointment must now face a more heavily filled appointment schedule. They may find it more difficult to book an appointment, which may help explain why waiting times are longer for such patients. This proposed mechanism is illustrated in the Figure.

**Figure.**
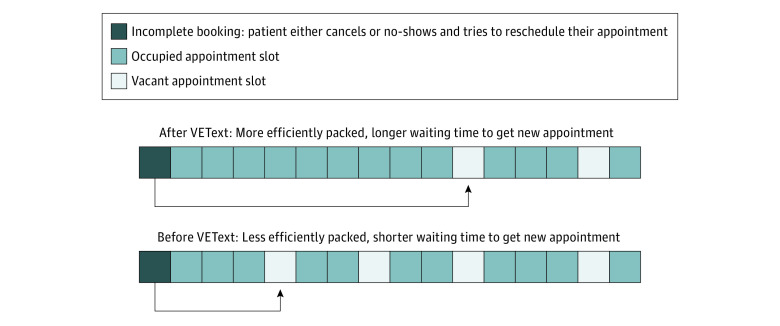
Proposed Mechanism Between Scheduling Efficiency and Waiting Time for a Patient With an Incomplete Booking VEText indicates electronic appointment reminder system via text messaging.

There are 2 major implications from these findings. First, the findings suggest that patient appointment-keeping behavior through electronic reminders may be associated with improved patient waiting times. With developments in digital and mobile technology, the costs of procuring and implementing such reminder systems have decreased, which may make it easier for clinics to implement them. Our findings suggest that implementing such systems can be part of a multipronged strategy to improve patient access.

Second, our findings suggest that improvement in timely access to care is not uniformly experienced by all patients; patients who have 1 or more incomplete bookings end up with longer waiting times under the new reminder system. This situation is a tradeoff that clinics make when their appointment books are filled more efficiently. Although this tradeoff is inevitable, clinics should nonetheless pay attention to such patients who have incomplete bookings and ensure that their waiting times, though lengthened, should not be unnecessarily long. This approach would be especially salient for patients who have serial or multiple incomplete bookings.

### Strengths and Limitations

One strength of this study is that it uses a large comprehensive data set of all appointments made at VA facilities across the US, which is representative of health services delivered through the VA. A limitation of this study is that it only uses data from VA health care facilities. Our findings may not be directly applicable to other organizations that deliver health care to the general US population. Furthermore, our analysis uses observational data, which has well-known limitations compared with randomized clinical trials. Nevertheless, we endeavored to address potential limitations by using standard statistical techniques (instrumental variable regression). In addition, the time window spanned by our data are relatively short (approximately 10 months). It is possible that the estimated associations between VEText and patient waiting times may differ over a longer time frame.

## Conclusions

Reducing the number of days patients wait for their appointments is an important goal that many clinical practices strive to meet. Electronic reminder systems, should, in principle, help patients to keep their appointments, but the consequences associated with patient waiting time may not be obvious. Using data from the VA health system, this study found that the implementation of the VEText reminder system was associated with a reduction in the mean waiting time for patients to complete their appointments. This finding suggests that such technology, when used systematically, can be a useful tool in helping to improve processes of health care delivery.

## Notes

Supplement. **eMethods.** Diagrams, Models, and Analyses **eTable 1.** Control Variables Used in Models 1-4 of Primary Analysis **eTable 2.** Full Results of Waiting Time Regression for Models 1-4 **eTable 3.** Main Waiting Time Regression Results From Models 3 and 4 With Progressively Added Controls **eTable 4.** Full Results Of Waiting Time Regression for Model 4, in Aggregate and Stratified by Clinical Group **eTable 5.** Summary of Results From Sensitivity Analyses **eTable 6.** Summary of Results From Additional Subgroup Analyses **eTable 7.** Full Regression Results for Number of Patient Cancellations (Post-Hoc Analysis), for all Clinical Groups and Stratified by Clinical Group **eTable 8.** Full Regression Results for Short Cancellations Within 21 Days (Post-Hoc Analysis), for all Clinical Groups and Stratified by Clinical Group **eTable 9.** Full Regression Results for Short Cancellations Within 14 Days (Post-Hoc Analysis), for all Clinical Groups and Stratified by Clinical Group **eTable 10.** Full Regression Results for Short Cancellations Within 7 Days (Post-Hoc Analysis), for all Clinical Groups and Stratified by Clinical Group
eReferences
