# Supplementary material for: Association of Use of Electronic Appointment Reminders With Waiting Times in the Veterans Affairs Health System
Source: JAMA Netw Open. 2022 Feb 15;5(2):e2148593. doi: 10.1001/jamanetworkopen.2021.48593 (PMC8848196; doi:10.1001/jamanetworkopen.2021.48593)
Supplement: Supplement. — eMethods. Diagrams, Models, and Analyses eTable 1. Control Variables Used in Models 1-4 of Primary Analysis eTable 2. Full Results of Waiting Time Regression for Models 1-4 eTable 3. Main Waiting Time Regression Results From Models 3 and 4 With Progressively Added Controls eTable 4. Full Results Of Waiting Time Regression for Model 4, in Aggregate and Stratified by Clinical Group eTable 5. Summary of Results From Sensitivity Analyses eTable 6. Summary of Results From Additional Subgroup Analyses eTable 7. Full Regression Results for Number of Patient Cancellations (Post-Hoc Analysis), for all Clinical Groups and Stratified by Clinical Group eTable 8. Full Regression Results for Short Cancellations Within 21 Days (Post-Hoc Analysis), for all Clinical Groups and Stratified by Clinical Group eTable 9. Full Regression Results for Short Cancellations Within 14 Days (Post-Hoc Analysis), for all Clinical Groups and Stratified by Clinical Group eTable 10. Full Regression Results for Short Cancellations Within 7 Days (Post-Hoc Analysis), for all Clinical Groups and Stratified by Clinical Group eReferences [file jamanetwopen-e2148593-s001.pdf]

Supplemental Online Content

Li L, Zhao H, Lim N, Goh J, Ng B. Association of use of electronic appointment reminders with waiting times in the Veterans Affairs Health System. *JAMA Netw Open.* 2022;5(2):e2148593. doi:10.1001/jamanetworkopen.2021.48593

eMethods. Diagrams, Models, and Analyses

eTable 1. Control Variables Used in Models 1-4 of Primary Analysis

eTable 2. Full Results of Waiting Time Regression for Models 1-4

eTable 3. Main Waiting Time Regression Results From Models 3 and 4 With Progressively Added Controls

eTable 4. Full Results Of Waiting Time Regression for Model 4, in Aggregate and Stratified by Clinical Group

eTable 5. Summary of Results From Sensitivity Analyses

eTable 6. Summary of Results From Additional Subgroup Analyses

eTable 7. Full Regression Results for Number of Patient Cancellations (Post-Hoc Analysis), for all Clinical Groups and Stratified by Clinical Group

eTable 8. Full Regression Results for Short Cancellations Within 21 Days (Post-Hoc Analysis), for all Clinical Groups and Stratified by Clinical Group

eTable 9. Full Regression Results for Short Cancellations Within 14 Days (Post-Hoc Analysis), for all Clinical Groups and Stratified by Clinical Group

eTable 10. Full Regression Results for Short Cancellations Within 7 Days (Post-Hoc Analysis), for all Clinical Groups and Stratified by Clinical Group

eReferences

This supplemental material has been provided by the authors to give readers additional information about their work.

© 2022 Li L et al. *JAMA Network Open*.

eMethods

Flow Diagram of Sample Construction

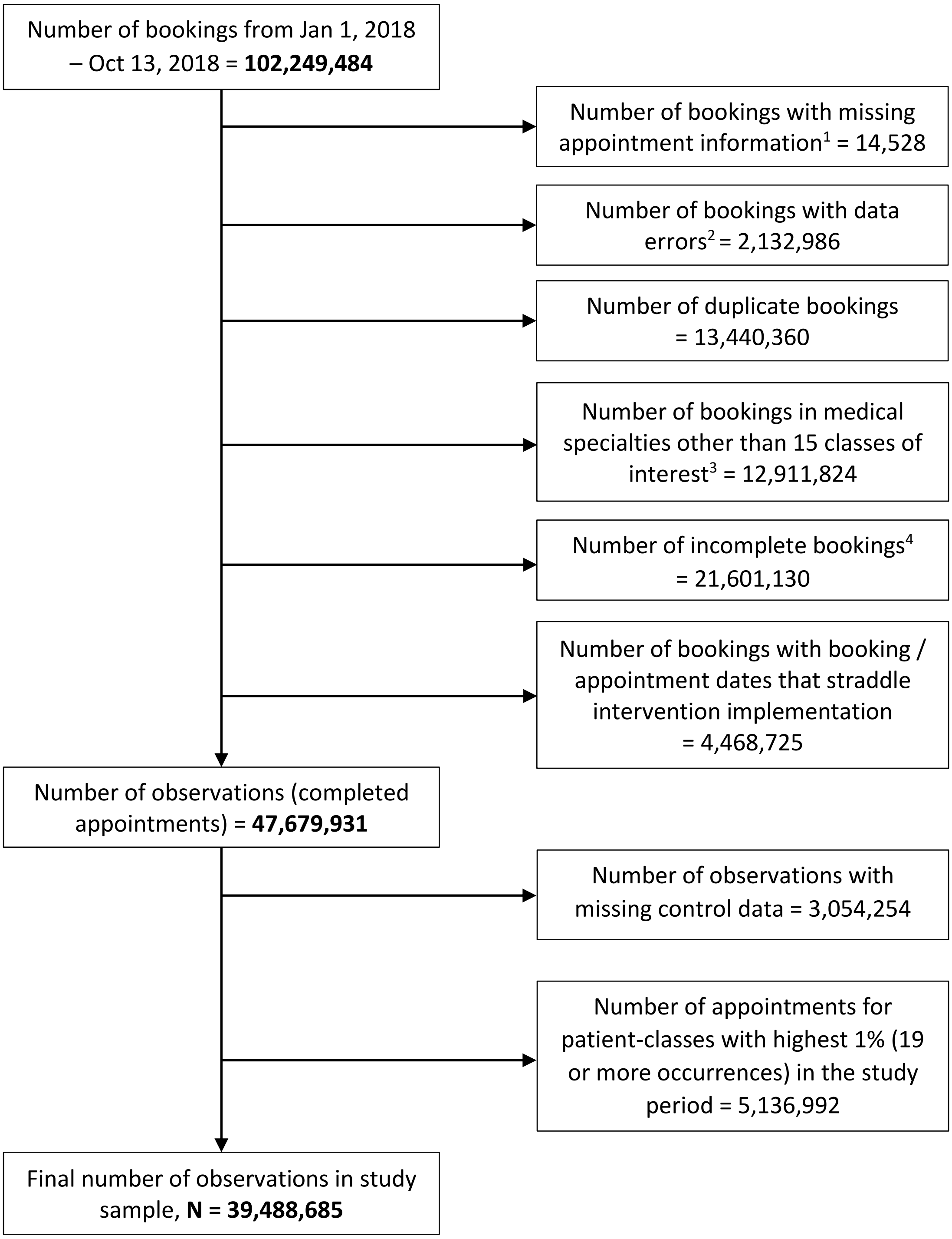

Notes:

[1] Missing booking dates, appointment dates, or appointment status

[2] Data errors include bookings where booking dates were after appointment dates

[3] Classes removed: emergency/urgent care, telephone clinics, home-based primary care, chaplain, social work

[4] Incomplete bookings refer to bookings that resulted in appointment cancellations or no-shows.

© 2022 Li L et al. *JAMA Network Open*.

Ordinary Linear Regression Models

We begin by describing Models 1 and 2 of our primary analysis of this paper, which are ordinary multivariable linear regressions. Mathematically, they are represented as follows:

Model 1: $$W_i = \beta_0 + \beta_T T_i + \boldsymbol{\beta}' \boldsymbol{X_i} + \epsilon_i$$

Model 2: $$W_i = \beta_0 + \beta_T T_i + \beta_B B_i + \beta_{T\times B}(T_i \times B_i) + \boldsymbol{\beta}' \boldsymbol{X_i} + \epsilon_i$$

In these expressions, scalar quantities are denoted in regular typeface, whereas vector quantities are denoted in boldface. The prime (‘) symbol represents vector transposition.

In both models, the symbol i is used to index all completed appointments, and the dependent variable, W_i, refers to the waiting time of the ith completed appointment, which is defined as the number of days between the date that the appointment is booked (booking date) and the date that the appointment is completed (appointment date).

The error term ϵ_i captures random noise that we assumed to be clustered at the clinic level, i.e., the level of the intervention variable. In other words, *within* all observations for a given clinic, arbitrary correlations were allowed in these terms. This noise model only assumed that these error terms were uncorrelated *across* different clinics. Allowing for this correlation typically leads to more conservative estimates (i.e., larger standard errors) than assuming that errors are independent across all observations.

The key independent variable in both models is the binary-valued *intervention indicator, T_i*. This takes the value $T_i = 0$ if the appointment date of the ith completed appointment comes before the clinic (at which the appointment was made) adopted VEText. Conversely, this takes the value $T_i = 1$ if the appointment date of the ith completed appointment falls after the clinic had adopted VEText and the patient had received at least one VEText reminder.

Model 2 includes another independent variable B_i, which refers to the number of incomplete bookings (i.e., were not completed due to cancellation or no-shows) associated with the completed appointment i. To be included in this count, the incomplete booking had to be made by the same patient for that same appointment class, and had to have a booking date that was between the booking date and appointment date of the completed appointment i.

Model 2 also contains an independent variable that is the interaction between the intervention indicator and the number of incomplete bookings, $T_i \times B_i$. We include this interaction to assess the change in association between the number of incomplete bookings and waiting time that is, in turn, associated with the VEText intervention. This comes from the estimated regression coefficient $\beta_{T\times B}$ because we can group terms as follows $\beta_B B_i + \beta_{T\times B}(T_i \times B_i) = (\beta_B + \beta_{T\times B} T_i)B_i$, from which this interpretation of the coefficient $\beta_{T\times B}$ should be more apparent.

In both models, the vector $\boldsymbol{X_i}$ represents the vector of controls associated with the ith completed appointment, which can be categorized into different sets of controls, listed in eTable 1. Full results for these regressions are reported in the first two columns of eTable 2.

Instrumental Variable Regression Models

As described in the main text, we implemented the instrumental variable (IV) regression models (Model 3 and Model 4) because individual clinics could choose when to adopt VEText, and this self-

© 2022 Li L et al. *JAMA Network Open*.

selection of the independent variable can result in bias in ordinary regression models. IV regression models were developed as a means of correcting such bias if an appropriate instrument can be found. The IV regression approach that we used is standard statistical procedure that is described and formally justified in many statistical textbooks.[1, 2] In what follows, we provide an overview of the approach as applied to our problem context.

The IV regression model specifications for Models 3 and 4 parallel those for Models 1 and 2 respectively. The key difference between the IV regressions and ordinary linear regressions is that each IV regression model involves estimating parameters for two sets of equations, which are respectively called the "selection equation(s)" and the "outcome equation". Model 3 has one selection equation and one outcome equation. Model 4 also has one outcome equation, but because this outcome equation includes an interaction term that involves the intervention indicator, we require two selection equations.[2, Chap 4.6.1]These are described below for each model.

Model 3:

$$T_i = \alpha_0 + \alpha_Z Z_i + \boldsymbol{\alpha}' \boldsymbol{X_i} + \nu_i \quad \text{(Selection Equation)}$$

$$W_i = \beta_0 + \beta_T \hat{T}_i + \boldsymbol{\beta}' \boldsymbol{X_i} + \epsilon_i \quad \text{(Outcome Equation)}$$

Model 4:

$$T_i = \alpha_0 + \alpha_Z Z_i + \alpha_{Z\times B}(Z_i \times B_i) + \boldsymbol{\alpha}' \boldsymbol{X_i} + \nu_i \quad \text{(Selection Equation 1)}$$

$$T_i \times B_i = \gamma_0 + \gamma_Z Z_i + \gamma_{Z\times B}(Z_i \times B_i) + \boldsymbol{\gamma}' \boldsymbol{X_i} + \eta_i \quad \text{(Selection Equation 2)}$$

$$W_i = \beta_0 + \beta_T \hat{T}_i + \beta_B B_i + \beta_{T\times B}\left(\widehat{T_i \times B_i}\right) + \boldsymbol{\beta}' \boldsymbol{X_i} + \epsilon_i \quad \text{(Outcome Equation)}$$

As with the ordinary linear regressions, the symbol i is used to index all completed appointments, and all other previously defined symbols retain their original definitions. We proceed to describe the new symbols introduced in these models.

The *instrumental variable* in both these models is represented by the binary-valued Z_i. This was assigned the value $Z_i = 1$ if the appointment date of the completed appointment i was scheduled on the third week or later of the centrally determined rollout date for that clinic, and $Z_i = 0$ otherwise. This was because each wave of the rollout was designated to have two full weeks of implementation.

The error term ν_i captures random noise in the first (and, in the case of Model 3, only) selection equation of each model. For Model 4, the error term η_i captures random noise in the second selection equation. Like the terms ϵ_i, both these error terms were assumed to be clustered at the clinic level.

From these models, the classical procedure for instrumental variable regression occurs in two steps.[2, Chap 4.6.1] We first describe the procedure for Model 4. First, estimates of the coefficients of the selection equations $(\hat{\alpha}_0, \hat{\alpha}_Z, \hat{\alpha}_{Z\times B}, \hat{\boldsymbol{\alpha}})$ and $(\hat{\gamma}_0, \hat{\gamma}_Z, \hat{\gamma}_{Z\times B}, \hat{\boldsymbol{\gamma}})$ are generated using ordinary linear regression. Next, using these coefficients, and for each observation, the *estimated intervention* $\hat{T}_i$ and the *estimated intervention interaction term* $\widehat{T_i \times B_i}$ are constructed using these estimated coefficients as follows:

$$\hat{T}_i = \hat{\alpha}_0 + \hat{\alpha}_Z Z_i + \hat{\alpha}_{Z\times B}(Z_i \times B_i) + \hat{\boldsymbol{\alpha}}' \boldsymbol{X_i}$$

$$\widehat{T_i \times B_i} = \hat{\gamma}_0 + \hat{\gamma}_z Z_i + \hat{\gamma}_{Z\times B}(Z_i \times B_i) + \hat{\boldsymbol{\gamma}}' \boldsymbol{X_i}$$

© 2022 Li L et al. *JAMA Network Open*.

In the second step, the dependent variable W_i is regressed now using the estimated intervention variable and interaction term $\hat{T}_i$ and $\widehat{T_i \times B_i}$ in place of the actual intervention variable and interaction term T_i and $T_i \times B_i$, with the other independent and control variables left unchanged. Through this process, the estimated coefficients from the second estimating equations of both models would then be properly adjusted for self-selection bias.

The procedure for Model 3 is analogous, except that there is only one selection equation. In the first step, only $(\hat{\alpha}_0, \hat{\alpha}_Z, \hat{\boldsymbol{\alpha}})$ is estimated. This is then used to construct the estimated intervention variable $\hat{T}_i$ in the second step.

Results for these regressions are reported in the rightmost two columns of eTable 2. In addition, eTable 3 presents the main results for these regressions as groups of control variables are progressively added into the model. Finally, for Model 4, which is the fully controlled model with incomplete bookings and interactions added as regressors, we report first-stage regression statistics as well as second-stage regression results in eTable 4, both in aggregate (first column) and by appointment groups (second to fifth column).

The first stage Kleibergen-Paap *rk* Lagrange Multiplier (LM) statistics indicate that the null hypothesis of underidentification should be rejected at the conventional 5% level, which provides evidence that the instrumental variable Z_i is indeed correlated with the treatment variable T_i.[3] Another validity threat to an IV regression can occur if this correlation between $Z_i$and T_i is non-zero, but weak (i.e., small in magnitude). Two statistical tests suggest that this is not the case in our setting: (a) The Kleibergen-Paap *rk* Wald *F*- statistics are substantially larger than the conventional rejection threshold of 10; and (b) the Cragg-Donald Wald *F*-statistics are also much larger than the Stock-Yogo critical values that conservatively assume a bias of 10%.[3, 4] Finally, with the exception of the Rehab group, the *C*-statistics indicate that the null hypothesis of treatment exogeneity should be rejected at the conventional 5% level.[3] Intuitively, these results mean that it would be inappropriate to perform ordinary linear regression by simply including the treatment T_i as a regressor. This further substantiates our use of a two-stage IV regression.

Regression Models for Post-hoc Analyses

In post-hoc analyses, we sought to investigate whether the introduction of VEText was associated with changes in the average number of cancellations per completed appointment. As described in the main text, we conducted these analyses to assess whether VEText might be plausibly associated with a more tightly packed appointment schedule, which would be likely if VEText was found to be associated with fewer cancellations.

These analyses were also conducted as IV regressions and follow the same two-step procedure described above. Their specifications parallel that of Model 3 in our primary analysis, and can be written as follows:

$$T_i = \alpha_0 + \alpha_Z Z_i + \boldsymbol{\alpha}' \boldsymbol{X_i} + \nu_i$$

$$C_i = \beta_0 + \beta_T \hat{T}_i + \boldsymbol{\beta}' \boldsymbol{X_i} + \epsilon_i$$

The unit of observation remains the same as in Model 3. The key difference stems from the dependent variable and this is reflected by our choice of a different symbol. In our main analysis, the dependent variable was waiting time, which we had denoted by W_i. In this post-hoc analysis, the dependent variable was the number of patient-cancelled bookings, which we denote by C_i. We conducted this regression analysis on the full sample, and repeated this analysis, stratified by clinical

© 2022 Li L et al. *JAMA Network Open*.

group. As described in the main text, we also conducted additional analyses where we separately investigated modifications of the dependent variable to the number of patient-cancelled bookings within 7, 14, and 21 days respectively.

Full results of these analyses are reported in eTables 7-10. These results reveal that the introduction of VEText was associated with a decrease in the average number of cancelled bookings, and average number of bookings that were cancelled at short notice, within 7, 14, and 21 days. These directional associations were found for all clinical groups in aggregate as well as for each clinical group.

Sensitivity and Subgroup Analyses

We conducted a suite of sensitivity and subgroup analyses to assess the robustness of the results to different model specifications and across different strata of the data. These were all based on IV regression Model 4, both across all clinical groups in aggregate and further stratified by clinical groups. We describe each of these analyses below.

Sensitivity Analyses

Sensitivity Analysis 1: In this group of analyses, we explored whether our results were robust when the maximum allowable waiting time was cut off at 12 months, 9 months, 6 months, and 3 months, respectively. We performed this analysis because it was possible that some appointments that were made far in advance could be routine appointments for which changes in waiting time would be less clinically meaningful.

Sensitivity Analysis 2: In the main regression analyses of our paper, the calendar month of the appointment was coded as a categorical variable. In this sensitivity analysis, we investigated how sensitive our results were to this specification, by changing month to a continuous variable.

Sensitivity Analysis 3: The IV approach used in our main regression analyses is technically called the standard two-stage least squares (2SLS) estimator, because it models both selection and outcome stages as linear regression problems. Although this approach has several appealing statistical properties, it is somewhat inefficient for our present problem because it doesn't exploit the fact that the treatment is binary-valued.

A known way to incorporate this information is by a simple modification of this setup that uses nonlinearly-fitted treatment probabilities to replace our original instrumental variables.[2, Chapter 4.6.1] To do this, we first construct a nonlinear regression model of the treatment on the instrument that exploits the binary nature of the data (such as a logistic or probit regression). For concreteness, we use the probit regression model specified below:

$$p_i \coloneqq Prob(T_i = 1)) = \Phi(\delta_0 + \delta_Z Z_i + \delta_{Z\times B}(Z_i \times B_i) + \boldsymbol{\delta}'\boldsymbol{X_i})$$

Where $\Phi(\cdot)$ represents the cumulative distribution function of the standard normal distribution. Maximum likelihood estimation is then used to estimate the coefficients of this regression $(\hat{\delta}_0, \hat{\delta}_Z, \hat{\delta}_{Z\times B}, \widehat{\boldsymbol{\delta}})$, from which we can obtain the fitted probabilities of assignment to treatment:

$$\hat{p}_i = \Phi(\hat{\delta}_0 + \hat{\delta}_Z Z_i + \hat{\delta}_{Z\times B}(Z_i \times B_i) + \widehat{\boldsymbol{\delta}}'\boldsymbol{X_i}).$$

Having obtained these fitted probabilities, we then implement the same 2SLS in our original approach, with the exception we use these fitted probabilities $\hat{p}_i$ in place of our original instrument Z_i.

The results of these analyses are summarized in eTable 5, which shows that the main results were mostly directionally robust to these alternative modeling assumptions and structures.

Subgroup Analyses

Subgroup Analysis 1: We conducted age-stratified analysis by dividing the sample into two age groups (65 and below vs 66 and above).

Subgroup Analysis 2: We conducted race-stratified analysis by dividing the sample into two patient groups (white vs non-white).

Subgroup Analysis 3*:* We stratified appointments based on whether private insurance was used for each given appointment. Private insurance was used in 68.1% of all completed appointments.

The results of these analyses are summarized in eTable 6. These findings are consistent with our study's primary findings: Across all strata, VEText was associated with a decrease in waiting time, each incomplete booking was associated with additional waiting time, and VEText was associated with an exacerbation of delay from incomplete bookings.

© 2022 Li L et al. *JAMA Network Open*.

eTable 1: *Control variables used in Models 1-4 of primary analysis.*

Control Type	Specific control variable
Patient's sociodemographic profile	Age at the time of the appointment, and square of the patient's age.<br><br>Gender (female, male).<br><br>Race/ethnicity (Asian, Afrian American/Black, American Indian, Pacific Islander, White.<br><br>Marital status (married, divorced, single, separated, widowed,).<br><br>Whether or not the patient used private insurance to supplement the financing of the appointment.
Seasonal / cyclical factors	Calendar month of the appointment, modelled as a categorical variable (levels: January, February, …, October).<br><br>Day of the week of the appointment, modelled as a categorical variable (levels: Sunday, Monday, …, Saturday).
Patient's utilization of VA health services	Total number of appointments made by that patient over the study period.<br><br>Total number of different medical classes that the patient had appointments with over the study period.
Clinic's congestion level	Total number of completed appointments in the same week.<br><br>Total number of booked appointments in the same week.<br><br>Total number of patients seen over the entire study period.
Individual facilities and appointment classes	Categorical variable indicating the VA health care facility for the appointment (130 levels in total, one for each facility).<br><br>Categorical variable indicating the medical class of the appointment (15 levels in total, one for each medical class).

© 2022 Li L et al. *JAMA Network Open*.

eTable 2: *Full results of waiting time regression for Models 1-4.*

Explanatory Variable	Model 1<br>Estimate (95% CI)	Model 2<br>Estimate (95% CI)	Model 3<br>Estimate (95% CI)	Model 4<br>Estimate (95% CI)
VEText intervention	-3.23 (-4.48 to -1.97)	-1.36 (-2.60 to -0.11)	-10.20 (-11.20 to -9.20)	-6.51 (-7.52 to -5.51)
Number of intermediate bookings		26.25 (25.40 to 27.10)		23.88 (23.11 to 24.66)
Interaction with VEText intervention		4.33 (3.25 to 5.42)		8.54 (7.65 to 9.44)
Age	1.34 (1.26 to 1.42)	1.39 (1.30 to 1.47)	1.34 (1.26 to 1.42)	1.39 (1.30 to 1.47)
Age^2	-0.009 (-0.009 to -0.008)	-0.009 (-0.010 to -0.008)	-0.009 (-0.009 to -0.008)	-0.009 (-0.010 to -0.008)
Sex				
Female (reference)				
Male	-0.13 (-0.68 to 0.42)	0.75 (0.21 to 1.30)	-0.15 (-0.70 to 0.40)	0.73 (0.19 to 1.28)
Race/Ethnicity				
White (reference)	---	---	---	---
African American / Black	-0.70 (-1.26 to -0.14)	-1.31 (-1.87 to -0.75)	-0.69 (-1.26 to -0.13)	-1.30 (-1.86 to -0.74)
Asian	2.10 (1.08 to 3.11)	2.53 (1.53 to 3.53)	2.10 (1.08 to 3.11)	2.54 (1.54 to 3.53)
American Indian	-0.74 (-1.12 to -0.35)	-1.24 (-1.62 to -0.86)	-0.74 (-1.13 to -0.36)	-1.24 (-1.62 to -0.86)
Pacific Islander	0.46 (-0.16 to 1.08)	0.04 (-0.57 to 0.64)	0.47 (-0.16 to 1.09)	0.04 (-0.56 to 0.64)
Marital status				
Married (reference)	---	---	---	---
Divorced	-2.52 (-2.89 to -2.15)	-3.02 (-3.38 to -2.65)	-2.53 (-2.90 to -2.16)	-3.01 (-3.38 to -2.65)
Single	-0.73 (-1.07 to -0.39)	-0.97 (-1.31 to -0.64)	-0.74 (-1.08 to -0.40)	-0.97 (-1.31 to -0.63)
Separated	-3.56 (-3.99 to -3.12)	-4.33 (-4.77 to -3.90)	-3.57 (-4.01 to -3.14)	-4.34 (-4.77 to -3.90)
Widowed	-2.90 (-3.35 to -2.45)	-3.32 (-3.77 to -2.88)	-2.92 (-3.37 to -2.47)	-3.33 (-3.78 to -2.88)
Private Insurance				
Used insurance for appointment	5.18 (4.85 to 5.50)	5.73 (5.39 to 6.07)	5.19 (4.87 to 5.52)	5.73 (5.40 to 6.07)
Calendar month of appointment date				
January (reference)	---	---	---	---

© 2022 Li L et al. *JAMA Network Open*.

February	4.75 (4.31 to 5.19)	2.16 (1.76 to 2.57)	4.76 (4.33 to 5.20)	2.37 (1.96 to 2.77)
March	7.27 (6.72 to 7.82)	3.59 (3.10 to 4.08)	7.48 (6.93 to 8.03)	4.01 (3.51 to 4.51)
April	9.07 (8.24 to 9.90)	5.52 (4.76 to 6.28)	10.86 (9.99 to 11.73)	6.89 (6.07 to 7.70)
May	8.52 (7.48 to 9.56)	5.06 (4.09 to 6.03)	12.55 (11.53 to 13.57)	7.70 (6.74 to 8.65)
June	10.32 (9.16 to 11.48)	5.82 (4.72 to 6.92)	15.43 (14.35 to 16.52)	9.00 (7.97 to 10.03)
July	12.08 (10.85 to 13.31)	6.51 (5.36 to 7.67)	17.46 (16.36 to 18.57)	9.79 (8.76 to 10.83)
August	14.35 (13.01 to 15.69)	8.14 (6.88 to 9.39)	19.99 (18.74 to 21.24)	11.53 (10.36 to 12.69)
September	17.16 (15.75 to 18.56)	10.54 (9.22 to 11.86)	22.94 (21.61 to 24.27)	13.99 (12.75 to 15.24)
October	16.29 (14.98 to 17.61)	9.36 (8.09 to 10.63)	22.17 (20.94 to 23.39)	12.84 (11.66 to 14.02)
Day of week of appointment date				
Sunday (reference)	---	---	---	---
Monday	13.46 (10.49 to 16.43)	12.82 (9.94 to 15.71)	13.42 (10.48 to 16.36)	12.79 (9.94 to 15.65)
Tuesday	11.13 (8.16 to 14.10)	10.40 (7.51 to 13.28)	11.09 (8.15 to 14.03)	10.36 (7.50 to 13.22)
Wednesday	10.64 (7.67 to 13.60)	9.81 (6.93 to 12.69)	10.61 (7.67 to 13.54)	9.78 (6.93 to 12.63)
Thursday	9.78 (6.82 to 12.74)	8.85 (5.98 to 11.73)	9.77 (6.84 to 12.70)	8.83 (5.99 to 11.68)
Friday	9.07 (6.11 to 12.03)	7.93 (5.05 to 10.80)	9.05 (6.12 to 11.98)	7.91 (5.06 to 10.75)
Saturday	1.59 (-2.85 to 6.03)	0.33 (-4.06 to 4.72)	1.54 (-2.89 to 5.97)	0.29 (-4.08 to 4.67)
Patient's utilization of VA health services				
Num. appointments	-0.12 (-0.13 to -0.10)	-0.18 (-0.20 to -0.17)	-0.12 (-0.13 to -0.11)	-0.18 (-0.20 to -0.17)
Num. classes	-1.16 (-1.40 to -0.92)	-1.00 (-1.23 to -0.76)	-1.16 (-1.40 to -0.92)	-1.00 (-1.24 to -0.76)
Clinic's congestion level				
Num. completed appointments in past week	-0.04 (-0.06 to -0.03)	-0.04 (-0.05 to -0.02)	-0.04 (-0.06 to -0.03)	-0.04 (-0.05 to -0.02)
Num. appointments in past week	0.03 (0.02 to 0.04)	0.03 (0.01 to 0.04)	0.03 (0.02 to 0.04)	0.02 (0.01 to 0.04)
Total number of patients seen (in 000s)	0.29 (0.08 to 0.51)	0.31 (0.09 to 0.53)	0.30 (0.08 to 0.51)	0.31 (0.09 to 0.53)
Constant term	12.76 (3.00 to 22.51)	6.80 (-2.65 to 16.25)	12.99 (2.98 to 22.99)	7.30 (-2.29 to 16.89)
Number of observations	39,488,685	39,488,685	39,488,685	39,488,685
R^2	0.102	0.161	0.101	0.160

Note: Coefficients for individual facility controls (130 levels) and appointment class controls (15 levels) omitted from table for brevity.

© 2022 Li L et al. *JAMA Network Open*.

eTable 3: *Main waiting time regression results from Models 3 and 4 with progressively added controls*

Explanatory Variable / Controls Included	(1)	(2)	(3)	(4)	(5)
**Main results from Model 3:**					
VEText intervention	6.73 (5.68 to 7.78)	7.13 (6.43 to 7.83)	-10.05 (-10.86 to -9.23)	-10.09 (-10.91 to -9.28)	-10.20 (-11.20 to -9.20)
Controls for facility and class	NO	YES	YES	YES	YES
Controls for month and day-of-week	NO	NO	YES	YES	YES
Controls for sociodemographic factors	NO	NO	NO	YES	YES
Controls for clinic-level congestion and patients' utilization of VA health services	NO	NO	NO	NO	YES
Number of observations	39,488,685	39,488,685	39,488,685	39,488,685	39,488,685
R^2	0.002	0.080	0.083	0.089	0.101
**Main results from Model 4:**					
VEText intervention	3.57 (2.60 to 4.53)	4.09 (3.50 to 4.69)	-6.51 (-7.34 to -5.68)	-6.50 (-7.32 to -5.68)	-6.51 (-7.52 to -5.51)
Number of intermediate bookings	23.04 (22.14 to 23.94)	23.48 (22.72 to 24.24)	23.07 (22.33 to 23.81)	23.40 (22.65 to 24.15)	23.88 (23.11 to 24.66)
Interaction with VEText intervention	9.26 (8.05 to 10.48)	8.43 (7.46 to 9.41)	8.60 (7.64 to 9.57)	8.66 (7.69 to 9.62)	8.54 (7.65 to 9.44)
Controls for facility and class	NO	YES	YES	YES	YES
Controls for month and day-of-week	NO	NO	YES	YES	YES
Controls for sociodemographic factors	NO	NO	NO	YES	YES
Controls for clinic-level congestion and patients' utilization of VA health services	NO	NO	NO	NO	YES
Number of observations	39,488,685	39,488,685	39,488,685	39,488,685	39,488,685
R^2	0.060	0.138	0.139	0.147	0.160

© 2022 Li L et al. *JAMA Network Open*.

eTable 4: *Full results of waiting time regression for Model 4, in aggregate and stratified by clinical group.*

Explanatory Variable	All Clinical Groups Estimate (95% CI)	Regular Outpatient Estimate (95% CI)	Procedural Estimate (95% CI)	Rehab Estimate (95% CI)	Radiology Estimate (95% CI)
**First stage statistics:**					
Under-identification test					
Kleibergen-Paap rk LM statistic	472.95	344.34	56.75	75.47	108.09
Chi-squared *p*-value	<0.001	<0.001	<0.001	<0.001	<0.001
Weak identification test					
Kleibergen-Paap rk Wald *F*-statistic	592.19	462.33	55.65	91.36	91.12
Cragg-Donald Wald *F*-statistic	2,285,163.66	2,228,270.74	223,451.35	129,528.89	63,494.66
Stock-Yogo critical values					
10% maximal IV size:	7.03	7.03	7.03	7.03	7.03
15% maximal IV size:	4.58	4.58	4.58	4.58	4.58
20% maximal IV size:	3.95	3.95	3.95	3.95	3.95
25% maximal IV size:	3.63	3.63	3.63	3.63	3.63
Endogeneity test					
C statistic	46.39	22.94	13.83	2.75	4.24
Chi-squared *p*-value	<0.001	<0.001	<0.001	0.0971	0.0395
**Second stage results:**					
VEText intervention	-6.51 (-7.52 to -5.51)	-5.73 (-7.04 to -4.41)	-10.24 (-13.64 to -6.84)	-1.52 (-2.17 to -0.86)	-3.31 (-4.98 to -1.64)
Number of intermediate bookings	23.88 (23.11 to 24.66)	25.21 (24.33 to 26.10)	22.73 (20.93 to 24.53)	9.34 (8.95 to 9.74)	15.71 (14.07 to 17.34)
Interaction with VEText intervention	8.54 (7.65 to 9.44)	7.80 (6.86 to 8.75)	15.15 (12.15 to 18.14)	2.32 (1.26 to 3.38)	16.62 (13.50 to 19.74)
Age	1.39 (1.30 to 1.47)	1.62 (1.52 to 1.71)	0.81 (0.64 to 0.98)	0.08 (0.04 to 0.12)	0.81 (0.70 to 0.92)
Age^2	-0.009 (-0.010 to -0.008)	-0.010 (-0.011 to -0.010)	-0.005 (-0.006 to -0.003)	-0.001 (-0.001 to -0.001)	-0.005 (-0.006 to -0.004)
Sex					
Female (reference)					
Male	0.73 (0.19 to 1.28)	1.13 (0.50 to 1.76)	1.18 (-0.10 to 2.46)	-0.50 (-0.73 to -0.27)	-1.42 (-2.54 to -0.30)

© 2022 Li L et al. *JAMA Network Open*.

Race/Ethnicity					
White (reference)	---	---	---	---	---
African American / Black	-1.30 (-1.86 to -0.74)	-1.46 (-2.12 to -0.79)	-1.43 (-2.68 to -0.19)	0.16 (-0.13 to 0.45)	-0.77 (-1.31 to -0.23)
Asian	2.54 (1.54 to 3.53)	2.95 (1.73 to 4.18)	1.51 (-0.10 to 3.13)	0.57 (0.05 to 1.08)	-0.39 (-1.04 to 0.25)
American Indian	-1.24 (-1.62 to -0.86)	-1.61 (-2.06 to -1.16)	-0.19 (-1.23 to 0.84)	0.02 (-0.38 to 0.42)	-1.05 (-1.68 to -0.43)
Pacific Islander	0.04 (-0.56 to 0.64)	0.15 (-0.63 to 0.94)	-0.48 (-1.63 to 0.67)	-0.22 (-0.73 to 0.29)	-0.67 (-1.28 to -0.06)
Marital status					
Married (reference)	---	---	---	---	---
Divorced	-3.01 (-3.38 to -2.65)	-3.46 (-3.90 to -3.01)	-3.17 (-3.91 to -2.43)	-0.51 (-0.64 to -0.38)	0.24 (0.02 to 0.45)
Single	-0.97 (-1.31 to -0.63)	-1.11 (-1.52 to -0.69)	-1.49 (-2.09 to -0.89)	-0.37 (-0.57 to -0.16)	0.77 (0.49 to 1.05)
Separated	-4.34 (-4.77 to -3.90)	-4.87 (-5.39 to -4.36)	-4.59 (-5.64 to -3.54)	-0.65 (-0.93 to -0.37)	0.15 (-0.21 to 0.50)
Widowed	-3.33 (-3.78 to -2.88)	-3.57 (-4.11 to -3.03)	-4.07 (-5.07 to -3.07)	-0.40 (-0.61 to -0.19)	0.06 (-0.32 to 0.44)
Private Insurance					
Used insurance for appointment	5.73 (5.40 to 6.07)	6.52 (6.13 to 6.92)	4.81 (3.98 to 5.64)	0.83 (0.68 to 0.98)	2.79 (2.41 to 3.17)
Calendar month of appointment date					
January (reference)	---	---	---	---	---
February	2.37 (1.96 to 2.77)	2.98 (2.41 to 3.54)	1.83 (1.00 to 2.67)	-1.14 (-1.42 to -0.86)	0.59 (0.10 to 1.09)
March	4.01 (3.51 to 4.51)	4.86 (4.22 to 5.50)	2.42 (1.14 to 3.70)	-0.80 (-1.14 to -0.46)	1.28 (0.70 to 1.86)
April	6.89 (6.07 to 7.70)	8.04 (6.96 to 9.13)	6.29 (4.32 to 8.26)	-0.15 (-0.61 to 0.31)	2.58 (1.85 to 3.32)
May	7.70 (6.74 to 8.65)	8.62 (7.31 to 9.94)	7.31 (4.99 to 9.64)	0.05 (-0.52 to 0.62)	3.15 (2.07 to 4.23)
June	9.00 (7.97 to 10.03)	9.37 (7.90 to 10.83)	8.73 (6.27 to 11.19)	0.89 (0.25 to 1.52)	4.01 (2.74 to 5.28)
July	9.79 (8.76 to 10.83)	9.90 (8.43 to 11.36)	10.81 (8.27 to 13.34)	2.08 (1.41 to 2.74)	4.03 (2.85 to 5.21)
August	11.53 (10.36 to 12.69)	12.28 (10.64 to 13.91)	11.79 (9.17 to 14.41)	1.78 (1.03 to 2.53)	3.64 (2.37 to 4.92)
September	13.99 (12.75 to 15.24)	14.76 (12.97 to 16.54)	15.11 (12.28 to 17.95)	2.43 (1.72 to 3.14)	5.21 (3.85 to 6.57)
October	12.84 (11.66 to 14.02)	12.99 (11.39 to 14.59)	16.01 (12.92 to 19.10)	2.25 (1.60 to 2.91)	5.92 (4.39 to 7.44)
Day of week of appointment date					
Sunday (reference)	---	---	---	---	---
Monday	12.79 (9.94 to 15.65)	23.73 (19.79 to 27.66)	-1.15 (-15.61 to 13.32)	2.15 (-4.90 to 9.19)	12.88 (10.11 to 15.65)
Tuesday	10.36 (7.50 to 13.22)	21.06 (17.13 to 24.99)	-3.57 (-18.12 to 10.97)	1.80 (-5.24 to 8.84)	10.89 (8.22 to 13.55)
Wednesday	9.78 (6.93 to 12.63)	20.63 (16.72 to 24.55)	-4.73 (-19.28 to 9.82)	1.63 (-5.40 to 8.66)	10.10 (7.40 to 12.79)
Thursday	8.83 (5.99 to 11.68)	19.46 (15.55 to 23.37)	-4.95 (-19.43 to 9.53)	1.25 (-5.79 to 8.30)	8.69 (6.11 to 11.27)

© 2022 Li L et al. *JAMA Network Open*.

Friday	7.91 (5.06 to 10.75)	18.09 (14.18 to 22.01)	-4.21 (-18.63 to 10.21)	0.73 (-6.36 to 7.81)	7.73 (5.14 to 10.31)
Saturday	0.29 (-4.08 to 4.67)	6.79 (0.45 to 13.13)	-4.10 (-20.61 to 12.42)	0.90 (-5.57 to 7.38)	2.42 (0.17 to 4.67)
Patient's utilization of VA health services					
Num. appointments	-0.18 (-0.20 to -0.17)	-0.20 (-0.22 to -0.19)	-0.25 (-0.31 to -0.19)	0.00 (0.00 to 0.01)	-0.04 (-0.05 to -0.03)
Num. classes	-1.00 (-1.24 to -0.76)	-0.85 (-1.13 to -0.58)	-1.84 (-2.45 to -1.23)	-0.09 (-0.16 to -0.03)	-0.75 (-0.89 to -0.60)
Clinic's congestion level					
Num. completed appts in past week	-0.04 (-0.05 to -0.02)	-0.06 (-0.08 to -0.04)	-0.03 (-0.05 to -0.01)	-0.01 (-0.02 to 0.00)	-0.26 (-0.32 to -0.20)
Num. appointments in past week	0.02 (0.01 to 0.04)	0.04 (0.03 to 0.05)	0.02 (0.00 to 0.03)	0.01 (0.00 to 0.02)	0.23 (0.17 to 0.29)
Total number of patients seen (in 000s)	0.31 (0.09 to 0.53)	0.14 (-0.08 to 0.37)	1.20 (0.92 to 1.48)	-0.20 (-0.55 to 0.14)	-0.76 (-1.81 to 0.30)
Constant term	7.30 (-2.29 to 16.89)	-5.83 (-15.95 to 4.29)	21.56 (0.32 to 42.81)	18.47 (9.44 to 27.50)	-28.86 (-35.12 to -22.60)
Number of observations	39,488,685	28,550,739	6,858,327	1,705,387	2,374,232
R^2	0.160	0.143	0.201	0.178	0.107

Note: Coefficients for individual facility controls (130 levels) and appointment class controls (15 levels) omitted from table for brevity.

© 2022 Li L et al. *JAMA Network Open*.

eTable 5: *Summary of results from sensitivity analyses.*

Explanatory Variable	All Clinical Groups<br>Estimate (95% CI)	Regular Outpatient<br>Estimate (95% CI)	Procedural<br>Estimate (95% CI)	Rehab<br>Estimate (95% CI)	Radiology<br>Estimate (95% CI)
**Sensitivity Analysis1: Only retain waiting time ≤ cutoff**					
Cutoff: 12 months					
VEText intervention	-5.45 (-6.36 to -4.53)	-4.69 (-5.88 to -3.49)	-9.06 (-12.38 to -5.74)	-1.45 (-2.10 to -0.80)	-2.55 (-4.14 to -0.96)
Number of incomplete bookings	20.73 (20.13 to 21.33)	21.83 (21.13 to 22.53)	19.55 (18.20 to 20.90)	9.19 (8.81 to 9.58)	14.10 (12.64 to 15.56)
Interaction with VEText intervention	5.97 (5.30 to 6.65)	5.36 (4.64 to 6.08)	10.65 (8.60 to 12.70)	2.25 (1.23 to 3.28)	14.31 (11.63 to 16.99)
Number of observations	39,069,729	28,180,615	6,815,231	1,705,117	2,368,766
Cutoff: 9 months					
VEText intervention	-4.26 (-4.92 to -3.60)	-3.94 (-4.83 to -3.05)	-5.25 (-7.41 to -3.09)	-1.40 (-2.04 to -0.76)	-1.71 (-3.08 to -0.33)
Number of incomplete bookings	20.21 (19.67 to 20.75)	21.34 (20.73 to 21.96)	18.80 (17.62 to 19.98)	9.14 (8.76 to 9.52)	13.66 (12.31 to 15.01)
Interaction with VEText intervention	4.74 (4.17 to 5.32)	4.22 (3.61 to 4.84)	8.53 (6.75 to 10.31)	2.14 (1.16 to 3.12)	13.08 (10.62 to 15.53)
Number of observations	38,182,221	27,564,232	6,568,341	1,704,789	2,344,859
Cutoff: 6 months					
VEText intervention	-3.03 (-3.54 to -2.53)	-3.01 (-3.65 to -2.37)	-2.87 (-4.85 to -0.89)	-1.27 (-1.90 to -0.65)	-1.37 (-2.90 to 0.15)
Number of incomplete bookings	15.76 (15.45 to 16.08)	16.57 (16.20 to 16.93)	14.37 (13.71 to 15.04)	9.18 (8.79 to 9.57)	12.26 (11.19 to 13.34)
Interaction with VEText intervention	2.65 (2.27 to 3.03)	2.33 (1.92 to 2.73)	4.78 (3.54 to 6.02)	1.23 (0.45 to 2.00)	8.61 (6.91 to 10.32)
Number of observations	35,605,890	25,394,051	6,198,081	1,702,413	2,311,345
Cutoff: 3 months					
VEText intervention	-0.91 (-1.20 to -0.62)	-0.83 (-1.17 to -0.49)	-1.13 (-1.86 to -0.40)	-0.22 (-0.76 to 0.32)	0.09 (-1.08 to 1.27)
Number of incomplete bookings	10.37 (10.18 to 10.55)	10.53 (10.30 to 10.76)	10.66 (10.24 to 11.08)	8.01 (7.67 to 8.35)	10.00 (9.44 to 10.56)
Interaction with VEText intervention	0.74 (0.55 to 0.94)	0.62 (0.39 to 0.84)	1.42 (0.63 to 2.21)	0.36 (0.08 to 0.64)	2.30 (1.31 to 3.30)
Number of observations	30,987,248	21,678,142	5,406,399	1,683,544	2,219,163
**Sensitivity Analysis 2: Calendar month as a continuous variable**					
VEText intervention	-6.17 (-6.93 to -5.40)	-5.98 (-6.98 to -4.98)	-10.04 (-12.86 to -7.23)	-0.75 (-1.28 to -0.21)	-2.30 (-3.67 to -0.94)
Number of incomplete bookings	23.99 (23.21 to 24.76)	25.37 (24.48 to 26.26)	22.72 (20.93 to 24.50)	9.31 (8.91 to 9.71)	15.74 (14.10 to 17.38)
Interaction with VEText intervention	8.39 (7.51 to 9.27)	7.56 (6.64 to 8.49)	15.16 (12.19 to 18.14)	2.39 (1.33 to 3.45)	16.59 (13.47 to 19.70)
Number of observations	39,488,685	28,550,739	6,858,327	1,705,387	2,374,232

© 2022 Li L et al. *JAMA Network Open*.

**Sensitivity Analysis 3: Using nonlinear fitted probabilities as instruments**					
VEText intervention	-5.68 (-7.87 to -3.49)	-3.78 (-6.82 to -0.74)	-9.40 (-12.94 to -5.87)	-1.87 (-3.13 to -0.61)	-3.58 (-5.22 to -1.94)
Number of incomplete bookings	24.07 (23.32 to 24.83)	25.39 (24.50 to 26.27)	23.13 (20.43 to 25.84)	9.85 (9.14 to 10.57)	19.59 (18.43 to 20.76)
Interaction with VEText intervention	8.22 (7.02 to 9.43)	7.56 (6.28 to 8.84)	14.31 (7.36 to 21.26)	1.36 (-0.08 to 2.81)	6.49 (4.27 to 8.71)
Number of observations	39,488,685	28,550,739	6,858,327	1,705,387	2,374,232

© 2022 Li L et al. *JAMA Network Open*.

eTable 6: *Summary of results from additional subgroup analyses.*

Explanatory Variable	All Clinical Groups Estimate (95% CI)	Regular Outpatient Estimate (95% CI)	Procedural Estimate (95% CI)	Rehab Estimate (95% CI)	Radiology Estimate (95% CI)
**Subgroup Analysis 1: Stratified by age group**					
Stratum 1: Age ≤ 65					
VEText intervention	-7.11 (-8.02 to -6.20)	-6.77 (-7.91 to -5.64)	-10.64 (-13.92 to -7.37)	-1.83 (-2.53 to -1.13)	-1.96 (-3.63 to -0.30)
Number of incomplete bookings	21.30 (20.46 to 22.14)	22.40 (21.42 to 23.38)	20.41 (18.33 to 22.50)	9.16 (8.75 to 9.57)	15.56 (14.70 to 16.43)
Interaction with VEText intervention	7.36 (6.30 to 8.42)	6.61 (5.46 to 7.77)	13.79 (10.23 to 17.35)	2.92 (1.97 to 3.87)	15.59 (13.00 to 18.19)
Number of observations	18,384,868	13,266,821	3,043,841	935,674	1,138,532
Stratum 2: Age > 65					
VEText intervention	-6.06 (-7.29 to -4.84)	-4.94 (-6.56 to -3.31)	-9.68 (-13.48 to -5.89)	-1.21 (-2.02 to -0.39)	-4.62 (-6.74 to -2.50)
Number of incomplete bookings	27.10 (26.35 to 27.86)	28.73 (27.89 to 29.56)	25.34 (23.61 to 27.08)	9.58 (9.07 to 10.09)	15.84 (12.99 to 18.69)
Interaction with VEText intervention	10.18 (9.34 to 11.02)	9.54 (8.71 to 10.36)	16.51 (13.55 to 19.47)	1.49 (-0.02 to 2.99)	17.71 (13.23 to 22.18)
Number of observations	21,103,817	15,283,918	3,814,486	769,713	1,235,700
**Sensitivity Analysis 2: Stratified by race**					
Stratum 1: White					
VEText intervention	-6.53 (-7.62 to -5.44)	-5.51 (-6.93 to -4.10)	-10.66 (-14.37 to -6.96)	-1.43 (-2.10 to -0.75)	-3.85 (-5.76 to -1.94)
Number of incomplete bookings	24.70 (23.91 to 25.50)	26.08 (25.18 to 26.99)	23.51 (21.64 to 25.37)	9.19 (8.72 to 9.65)	16.03 (13.98 to 18.07)
Interaction with VEText intervention	9.14 (8.30 to 9.98)	8.20 (7.32 to 9.07)	16.23 (13.17 to 19.29)	2.90 (2.04 to 3.77)	16.25 (12.90 to 19.59)
Number of observations	30,133,197	21,641,015	5,484,953	1,206,901	1,800,328
Stratum 2: Non-white					
VEText intervention	-6.75 (-7.86 to -5.65)	-6.74 (-8.16 to -5.32)	-8.60 (-11.91 to -5.29)	-1.89 (-2.97 to -0.80)	-2.14 (-4.63 to 0.35)
Number of incomplete bookings	21.78 (20.82 to 22.74)	23.02 (21.90 to 24.13)	20.26 (17.91 to 22.61)	9.66 (9.11 to 10.21)	14.88 (13.30 to 16.46)
Interaction with VEText intervention	7.01 (5.58 to 8.45)	6.78 (5.23 to 8.33)	11.46 (6.67 to 16.26)	1.22 (-1.08 to 3.51)	17.61 (13.09 to 22.14)
Number of observations	9,355,488	6,909,724	1,373,374	498,486	573,904
**Sensitivity Analysis 3: Stratified by usage of private insurance**					
Stratum 1: Used private insurance					
VEText intervention	-6.18 (-7.29 to -5.07)	-5.30 (-6.76 to -3.84)	-9.69 (-13.30 to -6.08)	-1.43 (-2.16 to -0.71)	-4.12 (-6.02 to -2.21)
Number of incomplete bookings	25.71 (24.93 to 26.49)	27.19 (26.31 to 28.07)	24.19 (22.32 to 26.05)	9.54 (9.13 to 9.96)	16.69 (14.50 to 18.88)
Interaction with VEText intervention	9.22 (8.37 to 10.07)	8.45 (7.59 to 9.30)	16.88 (13.88 to 19.88)	1.74 (0.29 to 3.19)	17.45 (13.83 to 21.07)

© 2022 Li L et al. *JAMA Network Open*.

Number of observations	27,161,996	19,669,053	4,778,103	1,125,850	1,588,990
Stratum 2: No private insurance					
VEText intervention	-7.27 (-8.19 to -6.34)	-6.73 (-7.88 to -5.58)	-11.51 (-14.82 to -8.21)	-1.71 (-2.58 to -0.84)	-1.73 (-3.64 to 0.17)
Number of incomplete bookings	21.01 (20.20 to 21.82)	22.08 (21.14 to 23.02)	20.41 (18.45 to 22.37)	8.97 (8.38 to 9.57)	14.19 (12.84 to 15.54)
Interaction with VEText intervention	7.31 (6.17 to 8.44)	6.66 (5.43 to 7.89)	11.88 (7.87 to 15.89)	3.38 (2.38 to 4.37)	15.12 (11.32 to 18.92)
Number of observations	12,326,689	8,881,686	2,080,224	579,537	785,242

© 2022 Li L et al. *JAMA Network Open*.

eTable 7: *Full regression results for number of patient cancellations (post-hoc analysis), for all clinical groups and stratified by clinical group.*

Explanatory Variable	All Clinical Groups Estimate (95% CI)	Regular Outpatient Estimate (95% CI)	Procedural Estimate (95% CI)	Rehab Estimate (95% CI)	Radiology Estimate (95% CI)
VEText intervention	-0.10 (-0.10 to -0.10)	-0.11 (-0.12 to -0.11)	-0.06 (-0.07 to -0.05)	-0.06 (-0.07 to -0.05)	-0.06 (-0.07 to -0.05)
Age	0.00 (0.00 to 0.00)	0.00 (0.00 to 0.00)	0.00 (0.00 to 0.00)	0.00 (0.00 to 0.00)	0.00 (0.00 to 0.00)
Age^2	0.00 (0.00 to 0.00)	0.00 (0.00 to 0.00)	0.00 (0.00 to 0.00)	0.00 (0.00 to 0.00)	0.00 (0.00 to 0.00)
Sex					
Female (reference)	---	---	---	---	---
Male	-0.04 (-0.04 to -0.04)	-0.04 (-0.04 to -0.04)	-0.02 (-0.02 to -0.02)	-0.05 (-0.05 to -0.04)	-0.04 (-0.04 to -0.03)
Race/Ethnicity					
White (reference)	---	---	---	---	---
African American / Black	0.00 (0.00 to 0.00)	0.00 (0.00 to 0.00)	0.00 (-0.01 to 0.00)	0.01 (0.01 to 0.01)	0.00 (0.00 to 0.00)
Asian	0.00 (0.00 to 0.00)	0.00 (0.00 to 0.00)	0.00 (-0.01 to 0.00)	0.00 (-0.01 to 0.01)	0.00 (0.00 to 0.01)
American Indian	0.01 (0.00 to 0.01)	0.01 (0.00 to 0.01)	0.00 (0.00 to 0.01)	0.01 (0.00 to 0.01)	0.00 (0.00 to 0.01)
Pacific Islander	0.01 (0.00 to 0.01)	0.01 (0.00 to 0.01)	0.00 (0.00 to 0.01)	0.01 (0.01 to 0.02)	0.00 (0.00 to 0.01)
Marital status					
Married (reference)	---	---	---	---	---
Divorced	0.00 (0.00 to 0.00)	0.00 (0.00 to 0.00)	0.00 (0.00 to 0.00)	0.00 (0.00 to 0.00)	0.01 (0.01 to 0.01)
Single	0.00 (0.00 to 0.00)	0.00 (0.00 to 0.00)	0.00 (0.00 to 0.00)	-0.01 (-0.01 to -0.01)	0.01 (0.00 to 0.01)
Separated	0.01 (0.00 to 0.01)	0.01 (0.00 to 0.01)	0.00 (0.00 to 0.01)	0.00 (0.00 to 0.01)	0.01 (0.01 to 0.01)
Widowed	0.00 (0.00 to 0.00)	0.00 (0.00 to 0.00)	0.00 (0.00 to 0.00)	0.00 (-0.01 to 0.00)	0.01 (0.01 to 0.01)
Private Insurance					
Used insurance for appointment	-0.01 (-0.01 to -0.01)	-0.01 (-0.01 to -0.01)	-0.01 (-0.01 to 0.00)	0.00 (0.00 to 0.00)	-0.01 (-0.01 to -0.01)
Calendar month of appointment date					
January (reference)	---	---	---	---	---
February	0.05 (0.05 to 0.05)	0.06 (0.06 to 0.06)	0.03 (0.03 to 0.03)	0.04 (0.03 to 0.04)	0.03 (0.03 to 0.04)
March	0.07 (0.07 to 0.07)	0.08 (0.08 to 0.08)	0.04 (0.04 to 0.05)	0.05 (0.04 to 0.05)	0.04 (0.04 to 0.04)

© 2022 Li L et al. *JAMA Network Open*.

April	0.08 (0.07 to 0.08)	0.09 (0.09 to 0.09)	0.05 (0.04 to 0.05)	0.04 (0.04 to 0.05)	0.05 (0.04 to 0.05)
May	0.09 (0.09 to 0.09)	0.11 (0.10 to 0.11)	0.05 (0.05 to 0.06)	0.05 (0.04 to 0.06)	0.05 (0.04 to 0.06)
June	0.11 (0.11 to 0.12)	0.13 (0.13 to 0.14)	0.07 (0.06 to 0.07)	0.07 (0.06 to 0.08)	0.06 (0.05 to 0.07)
July	0.13 (0.13 to 0.13)	0.15 (0.15 to 0.16)	0.08 (0.07 to 0.08)	0.08 (0.07 to 0.09)	0.07 (0.06 to 0.07)
August	0.14 (0.14 to 0.15)	0.17 (0.16 to 0.17)	0.08 (0.08 to 0.09)	0.08 (0.07 to 0.09)	0.07 (0.07 to 0.08)
September	0.15 (0.15 to 0.16)	0.18 (0.17 to 0.18)	0.09 (0.08 to 0.10)	0.09 (0.08 to 0.10)	0.08 (0.07 to 0.09)
October	0.16 (0.15 to 0.16)	0.18 (0.17 to 0.19)	0.09 (0.08 to 0.10)	0.10 (0.09 to 0.11)	0.08 (0.07 to 0.09)
Day of week of appointment date					
Sunday (reference)	---	---	---	---	---
Monday	0.01 (0.00 to 0.02)	0.03 (0.02 to 0.05)	0.03 (-0.02 to 0.08)	0.06 (0.01 to 0.12)	0.00 (0.00 to 0.01)
Tuesday	0.01 (0.01 to 0.02)	0.03 (0.02 to 0.05)	0.03 (-0.02 to 0.08)	0.07 (0.01 to 0.12)	0.01 (0.00 to 0.01)
Wednesday	0.01 (0.01 to 0.02)	0.04 (0.02 to 0.05)	0.03 (-0.01 to 0.08)	0.07 (0.01 to 0.12)	0.01 (0.00 to 0.01)
Thursday	0.02 (0.01 to 0.02)	0.04 (0.02 to 0.05)	0.03 (-0.01 to 0.08)	0.07 (0.01 to 0.12)	0.01 (0.00 to 0.01)
Friday	0.02 (0.01 to 0.03)	0.04 (0.03 to 0.06)	0.04 (-0.01 to 0.09)	0.07 (0.02 to 0.13)	0.01 (0.00 to 0.02)
Saturday	0.02 (0.01 to 0.03)	0.04 (0.02 to 0.06)	0.05 (0.01 to 0.10)	0.07 (0.00 to 0.13)	0.01 (0.00 to 0.01)
Patient's utilization of VA health services					
Num. appointments	0.00 (0.00 to 0.00)	0.00 (0.00 to 0.00)	0.00 (0.00 to 0.00)	0.00 (0.00 to 0.00)	0.00 (0.00 to 0.00)
Num. classes	0.00 (0.00 to 0.00)	0.00 (0.00 to 0.00)	0.00 (-0.01 to 0.00)	0.00 (0.00 to 0.00)	0.00 (0.00 to 0.00)
Clinic's congestion level					
Num. completed appts in past week	0.00 (0.00 to 0.00)	0.00 (0.00 to 0.00)	0.00 (0.00 to 0.00)	0.00 (0.00 to 0.00)	0.00 (0.00 to 0.00)
Num. appointments in past week	0.00 (0.00 to 0.00)	0.00 (0.00 to 0.00)	0.00 (0.00 to 0.00)	0.00 (0.00 to 0.00)	0.00 (0.00 to 0.00)
Total number of patients seen (in 000s)	0.00 (0.00 to 0.00)	0.00 (0.00 to 0.00)	0.00 (0.00 to 0.00)	-0.01 (-0.01 to 0.00)	0.00 (0.00 to 0.00)
Constant term	0.12 (0.10 to 0.13)	0.11 (0.09 to 0.13)	0.03 (-0.02 to 0.08)	0.13 (0.06 to 0.21)	-0.01 (-0.03 to 0.01)
Number of observations	39,488,685	28,550,739	6,858,327	1,705,387	2,374,232
R^2	0.012	0.012	0.008	0.012	0.008

Note: Coefficients for individual facility controls (130 levels) and appointment class controls (15 levels) omitted from table for brevity.

© 2022 Li L et al. *JAMA Network Open*.

eTable 8: *Full regression results for short cancellations within 21 days (post-hoc analysis), for all clinical groups and stratified by clinical group.*

Explanatory Variable	All Clinical Groups Estimate (95% CI)	Regular Outpatient Estimate (95% CI)	Procedural Estimate (95% CI)	Rehab Estimate (95% CI)	Radiology Estimate (95% CI)
VEText intervention	-0.09 (-0.09 to -0.08)	-0.10 (-0.10 to -0.09)	-0.05 (-0.06 to -0.05)	-0.06 (-0.07 to -0.05)	-0.06 (-0.07 to -0.05)
Age	0.00 (0.00 to 0.00)	0.00 (0.00 to 0.00)	0.00 (0.00 to 0.00)	0.00 (0.00 to 0.00)	0.00 (0.00 to 0.00)
Age^2	0.00 (0.00 to 0.00)	0.00 (0.00 to 0.00)	0.00 (0.00 to 0.00)	0.00 (0.00 to 0.00)	0.00 (0.00 to 0.00)
Sex					
Female (reference)	---	---	---	---	---
Male	-0.03 (-0.03 to -0.03)	-0.03 (-0.04 to -0.03)	-0.02 (-0.02 to -0.02)	-0.04 (-0.05 to -0.04)	-0.03 (-0.03 to -0.03)
Race/Ethnicity					
White (reference)	---	---	---	---	---
African American / Black	0.00 (0.00 to 0.00)	0.00 (0.00 to 0.00)	0.00 (0.00 to 0.00)	0.01 (0.01 to 0.02)	0.01 (0.00 to 0.01)
Asian	-0.01 (-0.01 to 0.00)	-0.01 (-0.01 to -0.01)	0.00 (-0.01 to 0.00)	0.00 (-0.01 to 0.00)	0.00 (-0.01 to 0.00)
American Indian	0.01 (0.01 to 0.01)	0.01 (0.01 to 0.01)	0.00 (0.00 to 0.01)	0.01 (0.00 to 0.01)	0.00 (0.00 to 0.01)
Pacific Islander	0.01 (0.00 to 0.01)	0.01 (0.00 to 0.01)	0.00 (0.00 to 0.00)	0.01 (0.00 to 0.02)	0.00 (0.00 to 0.01)
Marital status					
Married (reference)	---	---	---	---	---
Divorced	0.01 (0.01 to 0.01)	0.01 (0.01 to 0.01)	0.00 (0.00 to 0.01)	0.00 (0.00 to 0.00)	0.01 (0.01 to 0.01)
Single	0.00 (0.00 to 0.00)	0.00 (0.00 to 0.00)	0.00 (0.00 to 0.00)	-0.01 (-0.01 to 0.00)	0.01 (0.01 to 0.01)
Separated	0.01 (0.01 to 0.01)	0.01 (0.01 to 0.01)	0.01 (0.00 to 0.01)	0.00 (0.00 to 0.01)	0.01 (0.01 to 0.01)
Widowed	0.00 (0.00 to 0.01)	0.01 (0.00 to 0.01)	0.00 (0.00 to 0.00)	0.00 (-0.01 to 0.00)	0.01 (0.01 to 0.01)
Private Insurance					
Used insurance for appointment	-0.01 (-0.01 to -0.01)	-0.01 (-0.01 to -0.01)	-0.01 (-0.01 to -0.01)	0.00 (-0.01 to 0.00)	-0.01 (-0.01 to -0.01)
Calendar month of appointment date					
January (reference)	---	---	---	---	---
February	0.05 (0.04 to 0.05)	0.05 (0.05 to 0.05)	0.03 (0.03 to 0.03)	0.04 (0.03 to 0.04)	0.03 (0.03 to 0.04)
March	0.06 (0.06 to 0.07)	0.07 (0.07 to 0.07)	0.04 (0.03 to 0.04)	0.04 (0.04 to 0.05)	0.04 (0.04 to 0.04)
April	0.07 (0.06 to 0.07)	0.08 (0.07 to 0.08)	0.04 (0.03 to 0.04)	0.04 (0.03 to 0.04)	0.04 (0.04 to 0.05)
May	0.08 (0.07 to 0.08)	0.09 (0.09 to 0.09)	0.04 (0.04 to 0.05)	0.04 (0.04 to 0.05)	0.05 (0.04 to 0.05)

© 2022 Li L et al. *JAMA Network Open*.

June	0.10 (0.09 to 0.10)	0.11 (0.11 to 0.12)	0.06 (0.05 to 0.06)	0.06 (0.05 to 0.07)	0.05 (0.05 to 0.06)
July	0.11 (0.11 to 0.12)	0.13 (0.13 to 0.13)	0.06 (0.06 to 0.07)	0.07 (0.06 to 0.08)	0.06 (0.06 to 0.07)
August	0.12 (0.12 to 0.13)	0.14 (0.14 to 0.15)	0.07 (0.06 to 0.08)	0.08 (0.07 to 0.09)	0.07 (0.06 to 0.07)
September	0.13 (0.13 to 0.13)	0.15 (0.14 to 0.15)	0.07 (0.07 to 0.08)	0.09 (0.08 to 0.10)	0.07 (0.06 to 0.08)
October	0.13 (0.13 to 0.14)	0.15 (0.15 to 0.16)	0.08 (0.07 to 0.08)	0.09 (0.08 to 0.10)	0.07 (0.07 to 0.08)
Day of week of appointment date					
Sunday (reference)	---	---	---	---	---
Monday	0.01 (0.00 to 0.02)	0.03 (0.01 to 0.04)	0.02 (-0.03 to 0.07)	0.06 (0.00 to 0.12)	0.00 (0.00 to 0.01)
Tuesday	0.01 (0.00 to 0.02)	0.03 (0.02 to 0.05)	0.02 (-0.03 to 0.07)	0.06 (0.00 to 0.12)	0.00 (0.00 to 0.01)
Wednesday	0.01 (0.00 to 0.02)	0.03 (0.02 to 0.05)	0.02 (-0.03 to 0.07)	0.06 (0.00 to 0.12)	0.00 (0.00 to 0.01)
Thursday	0.01 (0.01 to 0.02)	0.03 (0.02 to 0.05)	0.02 (-0.03 to 0.07)	0.06 (0.00 to 0.12)	0.00 (0.00 to 0.01)
Friday	0.02 (0.01 to 0.03)	0.04 (0.02 to 0.05)	0.03 (-0.02 to 0.08)	0.07 (0.01 to 0.12)	0.01 (0.00 to 0.01)
Saturday	0.02 (0.01 to 0.03)	0.04 (0.02 to 0.05)	0.03 (-0.01 to 0.08)	0.06 (-0.01 to 0.12)	0.01 (0.00 to 0.01)
Patient's utilization of VA health services					
Num. appointments	0.00 (0.00 to 0.00)	0.00 (0.00 to 0.00)	0.00 (0.00 to 0.00)	0.00 (0.00 to 0.00)	0.00 (0.00 to 0.00)
Num. classes	0.00 (0.00 to 0.00)	0.00 (0.00 to 0.00)	0.00 (-0.01 to 0.00)	0.00 (0.00 to 0.00)	0.00 (0.00 to 0.00)
Clinic's congestion level					
Num. completed appts in past week	0.00 (0.00 to 0.00)	0.00 (0.00 to 0.00)	0.00 (0.00 to 0.00)	0.00 (0.00 to 0.00)	0.00 (0.00 to 0.00)
Num. appointments in past week	0.00 (0.00 to 0.00)	0.00 (0.00 to 0.00)	0.00 (0.00 to 0.00)	0.00 (0.00 to 0.00)	0.00 (0.00 to 0.00)
Total number of patients seen (in 000s)	0.00 (0.00 to 0.00)	0.00 (0.00 to 0.00)	0.00 (0.00 to 0.00)	-0.01 (-0.01 to 0.00)	0.00 (0.00 to 0.00)
Constant term	0.11 (0.10 to 0.12)	0.10 (0.08 to 0.12)	0.04 (-0.01 to 0.09)	0.13 (0.06 to 0.21)	0.00 (-0.02 to 0.02)
Number of observations	39,488,685	28,550,739	6,858,327	1,705,387	2,374,232
R^2	0.012	0.013	0.008	0.012	0.007

Note: Coefficients for individual facility controls (130 levels) and appointment class controls (15 levels) omitted from table for brevity.

© 2022 Li L et al. *JAMA Network Open*.

eTable 9: *Full regression results for short cancellations within 14 days (post-hoc analysis), for all clinical groups and stratified by clinical group.*

Explanatory Variable	All Clinical Groups Estimate (95% CI)	Regular Outpatient Estimate (95% CI)	Procedural Estimate (95% CI)	Rehab Estimate (95% CI)	Radiology Estimate (95% CI)
VEText intervention	-0.08 (-0.09 to -0.08)	-0.09 (-0.10 to -0.09)	-0.05 (-0.06 to -0.04)	-0.06 (-0.07 to -0.05)	-0.06 (-0.07 to -0.05)
Age	0.00 (0.00 to 0.00)	0.00 (0.00 to 0.00)	0.00 (0.00 to 0.00)	0.00 (0.00 to 0.00)	0.00 (0.00 to 0.00)
Age^2	0.00 (0.00 to 0.00)	0.00 (0.00 to 0.00)	0.00 (0.00 to 0.00)	0.00 (0.00 to 0.00)	0.00 (0.00 to 0.00)
Sex					
Female (reference)					
Male	-0.03 (-0.03 to -0.03)	-0.03 (-0.03 to -0.03)	-0.02 (-0.02 to -0.01)	-0.04 (-0.04 to -0.04)	-0.03 (-0.03 to -0.03)
Race/Ethnicity					
White (reference)	---	---	---	---	---
African American / Black	0.00 (0.00 to 0.00)	0.00 (0.00 to 0.00)	0.00 (0.00 to 0.00)	0.01 (0.01 to 0.02)	0.01 (0.00 to 0.01)
Asian	-0.01 (-0.01 to -0.01)	-0.01 (-0.01 to -0.01)	-0.01 (-0.01 to 0.00)	0.00 (-0.01 to 0.00)	0.00 (-0.01 to 0.00)
American Indian	0.01 (0.01 to 0.01)	0.01 (0.01 to 0.01)	0.01 (0.00 to 0.01)	0.01 (0.00 to 0.01)	0.01 (0.00 to 0.01)
Pacific Islander	0.01 (0.00 to 0.01)	0.01 (0.00 to 0.01)	0.00 (0.00 to 0.00)	0.01 (0.00 to 0.02)	0.00 (0.00 to 0.01)
Marital status					
Married (reference)	---	---	---	---	---
Divorced	0.01 (0.01 to 0.01)	0.01 (0.01 to 0.01)	0.00 (0.00 to 0.01)	0.00 (0.00 to 0.01)	0.01 (0.01 to 0.01)
Single	0.00 (0.00 to 0.00)	0.00 (0.00 to 0.00)	0.00 (0.00 to 0.00)	-0.01 (-0.01 to 0.00)	0.01 (0.01 to 0.01)
Separated	0.01 (0.01 to 0.01)	0.01 (0.01 to 0.01)	0.01 (0.00 to 0.01)	0.00 (0.00 to 0.01)	0.01 (0.01 to 0.01)
Widowed	0.01 (0.00 to 0.01)	0.01 (0.01 to 0.01)	0.00 (0.00 to 0.00)	0.00 (-0.01 to 0.00)	0.01 (0.01 to 0.01)
Private Insurance					
Used insurance for appointment	-0.01 (-0.01 to -0.01)	-0.01 (-0.01 to -0.01)	-0.01 (-0.01 to -0.01)	0.00 (-0.01 to 0.00)	-0.01 (-0.01 to -0.01)
Calendar month of appointment date					
January (reference)	---	---	---	---	---
February	0.04 (0.04 to 0.04)	0.05 (0.05 to 0.05)	0.03 (0.02 to 0.03)	0.04 (0.03 to 0.04)	0.03 (0.03 to 0.03)
March	0.06 (0.06 to 0.06)	0.07 (0.07 to 0.07)	0.04 (0.03 to 0.04)	0.04 (0.04 to 0.05)	0.04 (0.03 to 0.04)
April	0.06 (0.06 to 0.06)	0.07 (0.07 to 0.07)	0.04 (0.03 to 0.04)	0.04 (0.03 to 0.04)	0.04 (0.04 to 0.04)
May	0.07 (0.07 to 0.08)	0.08 (0.08 to 0.09)	0.04 (0.04 to 0.05)	0.04 (0.03 to 0.05)	0.04 (0.04 to 0.05)

© 2022 Li L et al. *JAMA Network Open*.

June	0.09 (0.09 to 0.09)	0.11 (0.10 to 0.11)	0.05 (0.05 to 0.06)	0.06 (0.05 to 0.07)	0.05 (0.05 to 0.06)
July	0.10 (0.10 to 0.11)	0.12 (0.12 to 0.13)	0.06 (0.05 to 0.06)	0.07 (0.06 to 0.08)	0.06 (0.05 to 0.06)
August	0.12 (0.11 to 0.12)	0.13 (0.13 to 0.14)	0.06 (0.06 to 0.07)	0.07 (0.06 to 0.08)	0.06 (0.06 to 0.07)
September	0.12 (0.12 to 0.13)	0.14 (0.14 to 0.15)	0.07 (0.06 to 0.07)	0.08 (0.07 to 0.09)	0.07 (0.06 to 0.07)
October	0.13 (0.12 to 0.13)	0.15 (0.14 to 0.15)	0.07 (0.06 to 0.08)	0.09 (0.08 to 0.10)	0.07 (0.06 to 0.08)
Day of week of appointment date					
Sunday (reference)	---	---	---	---	---
Monday	0.01 (0.00 to 0.02)	0.03 (0.01 to 0.04)	0.02 (-0.03 to 0.06)	0.05 (0.00 to 0.11)	0.00 (-0.01 to 0.01)
Tuesday	0.01 (0.00 to 0.02)	0.03 (0.01 to 0.04)	0.02 (-0.03 to 0.06)	0.06 (0.00 to 0.11)	0.00 (0.00 to 0.01)
Wednesday	0.01 (0.00 to 0.02)	0.03 (0.02 to 0.05)	0.02 (-0.03 to 0.07)	0.06 (0.00 to 0.11)	0.00 (0.00 to 0.01)
Thursday	0.01 (0.01 to 0.02)	0.03 (0.02 to 0.05)	0.02 (-0.03 to 0.07)	0.06 (0.00 to 0.11)	0.00 (0.00 to 0.01)
Friday	0.02 (0.01 to 0.02)	0.04 (0.02 to 0.05)	0.02 (-0.02 to 0.07)	0.06 (0.01 to 0.12)	0.01 (0.00 to 0.01)
Saturday	0.02 (0.01 to 0.02)	0.03 (0.02 to 0.05)	0.03 (-0.02 to 0.08)	0.06 (-0.01 to 0.12)	0.00 (0.00 to 0.01)
Patient's utilization of VA health services					
Num. appointments	0.00 (0.00 to 0.00)	0.00 (0.00 to 0.00)	0.00 (0.00 to 0.00)	0.00 (0.00 to 0.00)	0.00 (0.00 to 0.00)
Num. classes	0.00 (0.00 to 0.00)	0.00 (0.00 to 0.00)	0.00 (0.00 to 0.00)	0.00 (0.00 to 0.00)	0.00 (0.00 to 0.00)
Clinic's congestion level					
Num. completed appts in past week	0.00 (0.00 to 0.00)	0.00 (0.00 to 0.00)	0.00 (0.00 to 0.00)	0.00 (0.00 to 0.00)	0.00 (0.00 to 0.00)
Num. appointments in past week	0.00 (0.00 to 0.00)	0.00 (0.00 to 0.00)	0.00 (0.00 to 0.00)	0.00 (0.00 to 0.00)	0.00 (0.00 to 0.00)
Total number of patients seen (in 000s)	0.00 (0.00 to 0.00)	0.00 (0.00 to 0.00)	0.00 (0.00 to 0.00)	-0.01 (-0.01 to 0.00)	0.00 (0.00 to 0.00)
Constant term	0.11 (0.09 to 0.12)	0.10 (0.08 to 0.11)	0.05 (0.00 to 0.09)	0.13 (0.06 to 0.20)	0.00 (-0.02 to 0.03)
Number of observations	39,488,685	28,550,739	6,858,327	1,705,387	2,374,232
R^2	0.013	0.013	0.008	0.012	0.007

Note: Coefficients for individual facility controls (130 levels) and appointment class controls (15 levels) omitted from table for brevity.

© 2022 Li L et al. *JAMA Network Open*.

eTable 10: *Full regression results for short cancellations within 7 days (post-hoc analysis), for all clinical groups and stratified by clinical group.*

Explanatory Variable	All Clinical Groups Estimate (95% CI)	Regular Outpatient Estimate (95% CI)	Procedural Estimate (95% CI)	Rehab Estimate (95% CI)	Radiology Estimate (95% CI)
VEText intervention	-0.08 (-0.08 to -0.07)	-0.09 (-0.09 to -0.08)	-0.05 (-0.05 to -0.04)	-0.05 (-0.06 to -0.04)	-0.05 (-0.06 to -0.04)
Age	0.00 (0.00 to 0.00)	0.00 (0.00 to 0.00)	0.00 (0.00 to 0.00)	0.00 (0.00 to 0.00)	0.00 (0.00 to 0.00)
Age^2	0.00 (0.00 to 0.00)	0.00 (0.00 to 0.00)	0.00 (0.00 to 0.00)	0.00 (0.00 to 0.00)	0.00 (0.00 to 0.00)
Sex					
Female (reference)					
Male	-0.03 (-0.03 to -0.03)	-0.03 (-0.03 to -0.03)	-0.01 (-0.02 to -0.01)	-0.04 (-0.04 to -0.03)	-0.03 (-0.03 to -0.02)
Race/Ethnicity					
White (reference)	---	---	---	---	---
African American / Black	0.01 (0.01 to 0.01)	0.01 (0.01 to 0.01)	0.00 (0.00 to 0.01)	0.01 (0.01 to 0.02)	0.01 (0.01 to 0.01)
Asian	-0.01 (-0.01 to -0.01)	-0.01 (-0.01 to -0.01)	-0.01 (-0.01 to 0.00)	-0.01 (-0.01 to 0.00)	-0.01 (-0.01 to 0.00)
American Indian	0.01 (0.01 to 0.01)	0.01 (0.01 to 0.01)	0.01 (0.00 to 0.01)	0.01 (0.00 to 0.01)	0.01 (0.00 to 0.01)
Pacific Islander	0.01 (0.00 to 0.01)	0.01 (0.01 to 0.01)	0.00 (0.00 to 0.00)	0.01 (0.00 to 0.02)	0.00 (0.00 to 0.01)
Marital status					
Married (reference)	---	---	---	---	---
Divorced	0.01 (0.01 to 0.01)	0.01 (0.01 to 0.01)	0.01 (0.00 to 0.01)	0.00 (0.00 to 0.01)	0.01 (0.01 to 0.01)
Single	0.00 (0.00 to 0.00)	0.00 (0.00 to 0.00)	0.00 (0.00 to 0.00)	-0.01 (-0.01 to 0.00)	0.01 (0.01 to 0.01)
Separated	0.01 (0.01 to 0.01)	0.01 (0.01 to 0.01)	0.01 (0.01 to 0.01)	0.01 (0.00 to 0.01)	0.01 (0.01 to 0.02)
Widowed	0.01 (0.01 to 0.01)	0.01 (0.01 to 0.01)	0.00 (0.00 to 0.01)	0.00 (0.00 to 0.00)	0.01 (0.01 to 0.01)
Private Insurance					
Used insurance for appointment	-0.01 (-0.01 to -0.01)	-0.01 (-0.01 to -0.01)	-0.01 (-0.01 to -0.01)	0.00 (-0.01 to 0.00)	-0.01 (-0.01 to -0.01)
Calendar month of appointment date					
January (reference)	---	---	---	---	---
February	0.04 (0.04 to 0.04)	0.04 (0.04 to 0.04)	0.02 (0.02 to 0.02)	0.03 (0.03 to 0.04)	0.03 (0.03 to 0.03)
March	0.05 (0.05 to 0.05)	0.06 (0.06 to 0.06)	0.03 (0.03 to 0.03)	0.04 (0.03 to 0.04)	0.03 (0.03 to 0.04)
April	0.05 (0.05 to 0.06)	0.06 (0.06 to 0.06)	0.03 (0.03 to 0.03)	0.03 (0.03 to 0.04)	0.03 (0.03 to 0.04)
May	0.06 (0.06 to 0.07)	0.07 (0.07 to 0.08)	0.03 (0.03 to 0.04)	0.04 (0.03 to 0.05)	0.04 (0.03 to 0.04)

© 2022 Li L et al. *JAMA Network Open*.

June	0.08 (0.08 to 0.08)	0.09 (0.09 to 0.10)	0.04 (0.04 to 0.05)	0.05 (0.05 to 0.06)	0.04 (0.04 to 0.05)
July	0.09 (0.09 to 0.10)	0.11 (0.10 to 0.11)	0.05 (0.04 to 0.05)	0.06 (0.05 to 0.07)	0.05 (0.04 to 0.05)
August	0.10 (0.10 to 0.11)	0.12 (0.11 to 0.12)	0.05 (0.05 to 0.06)	0.07 (0.06 to 0.07)	0.05 (0.05 to 0.06)
September	0.11 (0.10 to 0.11)	0.12 (0.12 to 0.13)	0.06 (0.05 to 0.06)	0.08 (0.07 to 0.09)	0.06 (0.05 to 0.06)
October	0.11 (0.11 to 0.12)	0.13 (0.13 to 0.13)	0.06 (0.05 to 0.07)	0.08 (0.07 to 0.09)	0.06 (0.05 to 0.07)
Day of week of appointment date					
Sunday (reference)	---	---	---	---	---
Monday	0.01 (0.00 to 0.01)	0.03 (0.01 to 0.04)	0.02 (-0.03 to 0.06)	0.05 (0.00 to 0.10)	0.00 (-0.01 to 0.01)
Tuesday	0.01 (0.00 to 0.02)	0.03 (0.01 to 0.04)	0.02 (-0.03 to 0.06)	0.05 (0.00 to 0.10)	0.00 (0.00 to 0.01)
Wednesday	0.01 (0.00 to 0.02)	0.03 (0.01 to 0.04)	0.02 (-0.03 to 0.06)	0.05 (0.00 to 0.10)	0.00 (0.00 to 0.01)
Thursday	0.01 (0.00 to 0.02)	0.03 (0.02 to 0.04)	0.02 (-0.03 to 0.06)	0.05 (0.00 to 0.10)	0.00 (0.00 to 0.01)
Friday	0.01 (0.01 to 0.02)	0.03 (0.02 to 0.05)	0.02 (-0.02 to 0.07)	0.06 (0.01 to 0.11)	0.00 (0.00 to 0.01)
Saturday	0.01 (0.01 to 0.02)	0.03 (0.02 to 0.04)	0.03 (-0.02 to 0.07)	0.05 (-0.01 to 0.11)	0.00 (0.00 to 0.01)
Patient's utilization of VA health services					
Num. appointments	0.00 (0.00 to 0.00)	0.00 (0.00 to 0.00)	0.00 (0.00 to 0.00)	0.00 (0.00 to 0.00)	0.00 (0.00 to 0.00)
Num. classes	0.00 (0.00 to 0.00)	0.00 (0.00 to 0.00)	0.00 (0.00 to 0.00)	0.00 (0.00 to 0.00)	0.00 (0.00 to 0.00)
Clinic's congestion level					
Num. completed appts in past week	0.00 (0.00 to 0.00)	0.00 (0.00 to 0.00)	0.00 (0.00 to 0.00)	0.00 (0.00 to 0.00)	0.00 (0.00 to 0.00)
Num. appointments in past week	0.00 (0.00 to 0.00)	0.00 (0.00 to 0.00)	0.00 (0.00 to 0.00)	0.00 (0.00 to 0.00)	0.00 (0.00 to 0.00)
Total number of patients seen (in 000s)	0.00 (0.00 to 0.00)	0.00 (0.00 to 0.00)	0.00 (0.00 to 0.00)	-0.01 (-0.01 to 0.00)	0.00 (0.00 to 0.00)
Constant term	0.10 (0.09 to 0.11)	0.09 (0.07 to 0.11)	0.05 (0.00 to 0.09)	0.12 (0.06 to 0.19)	0.01 (-0.02 to 0.03)
Number of observations	39,488,685	28,550,739	6,858,327	1,705,387	2,374,232
R^2	0.013	0.013	0.008	0.013	0.007

Note: Coefficients for individual facility controls (130 levels) and appointment class controls (15 levels) omitted from table for brevity.

© 2022 Li L et al. *JAMA Network Open*.

eReferences

1. Greene WH. Econometric analysis 4th edition. International edition, New Jersey: Prentice Hall. 2000:201-15.
2. Angrist JD, Pischke J-S. Mostly harmless econometrics: An empiricist's companion: Princeton university press; 2008.
3. Baum CF, Schaffer ME, Stillman S. Enhanced routines for instrumental variables/generalized method of moments estimation and testing. Stata Journal. 2007;7(4):465-506.
4. Stock JH, Yogo M. Testing for Weak Instruments in Linear IV Regression. In: Andrews DWK, Stock JH, editors. Identification and Inference for Econometric Models: Essays in Honor of Thomas Rothenberg. Cambridge: Cambridge University Press; 2005. p. 80-108.

© 2022 Li L et al. *JAMA Network Open*.
